# Spatially resolved expression landscape and gene-regulatory network of human gastric corpus epithelium

**DOI:** 10.1093/procel/pwac059

**Published:** 2022-11-15

**Authors:** Ji Dong, Xinglong Wu, Xin Zhou, Yuan Gao, Changliang Wang, Wendong Wang, Weiya He, Jingyun Li, Wenjun Deng, Jiayu Liao, Xiaotian Wu, Yongqu Lu, Antony K Chen, Lu Wen, Wei Fu, Fuchou Tang

**Affiliations:** Biomedical Pioneering Innovation Center, Department of General Surgery, College of Life Sciences, Third Hospital, Peking University, Beijing 100871, China; GMU-GIBH Joint School of Life Sciences, Guangzhou Laboratory, Guangzhou Medical University, Guangzhou 510799, China; Bioland Laboratory (Guangzhou Regenerative Medicine and Health Guangdong Laboratory), Guangzhou 510320, China; College of Animal Science and Technology, Hebei Agricultural University, Baoding 071001, China; Biomedical Pioneering Innovation Center, Department of General Surgery, College of Life Sciences, Third Hospital, Peking University, Beijing 100871, China; Peking University Third Hospital Cancer Center, Beijing 100191, China; Biomedical Pioneering Innovation Center, Department of General Surgery, College of Life Sciences, Third Hospital, Peking University, Beijing 100871, China; Peking-Tsinghua Center for Life Sciences, Peking University, Beijing 100871, China; GMU-GIBH Joint School of Life Sciences, Guangzhou Laboratory, Guangzhou Medical University, Guangzhou 510799, China; Biomedical Pioneering Innovation Center, Department of General Surgery, College of Life Sciences, Third Hospital, Peking University, Beijing 100871, China; GMU-GIBH Joint School of Life Sciences, Guangzhou Laboratory, Guangzhou Medical University, Guangzhou 510799, China; Bioland Laboratory (Guangzhou Regenerative Medicine and Health Guangdong Laboratory), Guangzhou 510320, China; Biomedical Pioneering Innovation Center, Department of General Surgery, College of Life Sciences, Third Hospital, Peking University, Beijing 100871, China; GMU-GIBH Joint School of Life Sciences, Guangzhou Laboratory, Guangzhou Medical University, Guangzhou 510799, China; GMU-GIBH Joint School of Life Sciences, Guangzhou Laboratory, Guangzhou Medical University, Guangzhou 510799, China; Department of Biomedical Engineering, College of Engineering, Peking University, Beijing 100871, China; Biomedical Pioneering Innovation Center, Department of General Surgery, College of Life Sciences, Third Hospital, Peking University, Beijing 100871, China; Department of Biomedical Engineering, College of Engineering, Peking University, Beijing 100871, China; Biomedical Pioneering Innovation Center, Department of General Surgery, College of Life Sciences, Third Hospital, Peking University, Beijing 100871, China; Beijing Advanced Innovation Center for Genomics (ICG), Ministry of Education Key Laboratory of Cell Proliferation and Differentiation, Beijing 100871, China; Biomedical Pioneering Innovation Center, Department of General Surgery, College of Life Sciences, Third Hospital, Peking University, Beijing 100871, China; Peking University Third Hospital Cancer Center, Beijing 100191, China; Biomedical Pioneering Innovation Center, Department of General Surgery, College of Life Sciences, Third Hospital, Peking University, Beijing 100871, China; Beijing Advanced Innovation Center for Genomics (ICG), Ministry of Education Key Laboratory of Cell Proliferation and Differentiation, Beijing 100871, China; Peking-Tsinghua Center for Life Sciences, Peking University, Beijing 100871, China

**Keywords:** human gastric corpus, gastric corpus stem/progenitor cell, single-cell omics sequencing, single-cell ATAC-seq, spatial transcriptomics, regulatory network

## Abstract

Molecular knowledge of human gastric corpus epithelium remains incomplete. Here, by integrated analyses using single-cell RNA sequencing (scRNA-seq), spatial transcriptomics, and single-cell assay for transposase accessible chromatin sequencing (scATAC-seq) techniques, we uncovered the spatially resolved expression landscape and gene-regulatory network of human gastric corpus epithelium. Specifically, we identified a stem/progenitor cell population in the isthmus of human gastric corpus, where EGF and WNT signaling pathways were activated. Meanwhile, *LGR4*, but not *LGR5*, was responsible for the activation of WNT signaling pathway. Importantly, *FABP5* and *NME1* were identified and validated as crucial for both normal gastric stem/progenitor cells and gastric cancer cells. Finally, we explored the epigenetic regulation of critical genes for gastric corpus epithelium at chromatin state level, and identified several important cell-type-specific transcription factors. In summary, our work provides novel insights to systematically understand the cellular diversity and homeostasis of human gastric corpus epithelium *in vivo*.

## Introduction

Gastric cancer remains the fourth leading cause of cancer-related mortality worldwide due to limited treatment choices. Most gastric cancers develop through the Correa pathway, including gastritis, atrophy, intestinal metaplasia, dysplasia, and ultimately cancer (Correa, 1992; Correa and Shiao, 1994; Uemura et al., 2001). Recently, mouse model-based lineage-tracing studies and human sample-based clonality analyses demonstrated that the origins of both metaplasia and cancer tend to be tissue-resident stem cells (Hayakawa et al., 2017). Therefore, a better understanding of gastric stem/progenitor cells will help to better characterize gastric carcinogenesis and provide new treatment options.

Gastric stem cells play a crucial role in the lifelong self-renewal and homeostasis of the stomach. Although *Lgr5*-expressing cells represent the undisputed stem-cell population in small intestine, the identity of gastric stem cells is still controversial (Seidlitz et al., 2021). Several studies have been dedicated to find the representative marker genes of gastric stem cells (Qiao et al., 2007; Barker et al., 2010; Arnold et al., 2011; Hayakawa et al., 2015a, 2015b; Matsuo et al., 2016; Choi et al., 2018; Tan et al., 2020). In antral glands, genes such as *Villin*, *Lgr5*, *CCKR2*, *Sox2*, *eR1*, and *Aqp5* have been reported as candidate stem-cell markers, while in corpus glands, genes such as *Lrig1*, *Sox2*, *Troy*, and *Mist1* have been identified. However, these identified marker genes were not consistent across different studies. In addition, the studies to identify gastric stem cells were mainly based on mouse models, and to what extent mouse gastric stem cells resemble human gastric stem cells remains largely unknown.

In this work, we first utilized single-cell RNA sequencing (scRNA-seq) technique to profile the transcriptomes of human gastric corpus epithelial cells and recognize their identities based on the expression patterns. Importantly, we tried to identify the gastric stem/progenitor cells, which are still under debate. Then, we performed spatial transcriptomics analysis with the 10× Genomics Visium technique to map each cell population in the intact tissues and inferred the cell–cell interactions. Finally, we used single-cell assay for transposase accessible chromatin sequencing (scATAC-seq) technique to study the chromatin accessibility dynamics and gene-regulatory network of human gastric corpus epithelium. Our study systematically revealed the transcriptomic and epigenomic features of human gastric corpus epithelium at single-cell resolution, and would potentially be of great help for future studies of gastric diseases and gastric cancer treatment.

## Results

### Gene expression landscapes of human gastric corpus epithelium

**Figure 1.**
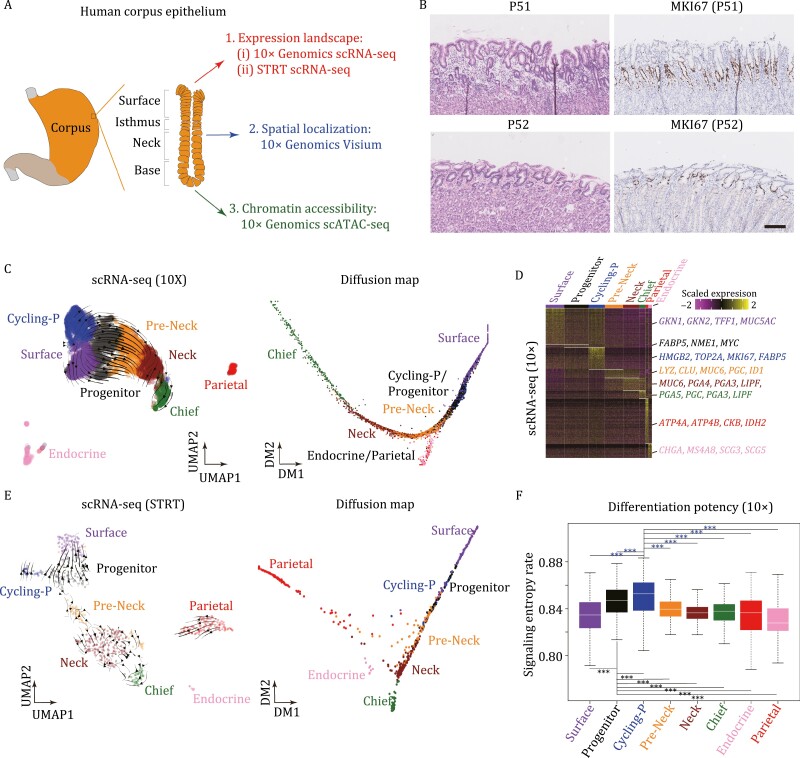
Gene expression landscapes of human gastric corpus epithelium. (A) Sketch of human gastric corpus tissue sampling and multi-omics profiling. Human gastric corpus samples were biopsied and sequenced using scRNA-seq, spatial transcriptomes, and scATAC-seq techniques. (B) H&E and MKI67 immunohistochemistry staining on gastric corpus samples of P51 and P52. Scale bar: 100 μm. (C) UMAP plot (left) and diffusion map plot (right) exhibiting the identified clusters of 10× dataset in human gastric corpus. Arrows in the UMAP plot indicate the differentiation routes inferred by RNA velocity analysis. Clusters are indicated by colors. (D) Heatmap exhibiting DEGs and representative marker genes of each cluster in gastric corpus 10× dataset. The color key from purple to yellow indicates low to high expression levels, respectively. (E) UMAP plot (left) and diffusion map plot (right) exhibiting the identified clusters of STRT dataset in gastric corpus. Arrows in the UMAP plot indicate the differentiation routes inferred by RNA velocity analysis. Clusters are indicated by colors. (F) Differentiation potency inferred through SCENT for each cluster in gastric corpus 10× dataset. Significance was determined by *t*-tests. *P-value <0.05, ***P-value <0.001.

To explore the cellular diversity of human gastric corpus epithelium, we sampled gastric corpus from two non-gastric cancer patients (i.e., P51, a pancreatic cancer patient, and P52, a right-sided colon cancer patient), who underwent pancreaticoduodectomy (due to the shared blood supply of the organs in the proximal gastrointestinal system, surgical removal of tumors also necessitates removal of the distal part of stomach) (Table S1). These gastric tissues were relatively normal compared with the adjacent normal tissues sampled from gastric cancer patients (Fig. 1A and 1B). We sequenced these cells using two mature scRNA-seq techniques: 10× Genomics and STRT (Islam et al., 2011; Dong et al., 2018), which could balance between throughput and accuracy. After stringent filtering, 9,229 cells for 10× and 1,172 cells for STRT were retained for subsequent analyses (Fig. S1A).

10×- and STRT-based datasets generated similar results and both captured eight clusters of cells through Seurat graph-based clustering after Harmony batch effect corrections (Figs. 1C, 1E, and S1B; Table S1) (see Methods) (Satija et al., 2015; Korsunsky et al., 2019). Based on the differentially expressed genes (DEGs) (Figs. 1D, S1C, and S1D; Table S2), we identified six of these eight clusters as differentiated cell types: surface mucous cells (with *GKN1* and *GKN2* specifically expressed), pre-mucous neck cells (*LYZ*, *CLU*, and *MUC6*), mucous neck cells (*MUC6*, *PGA4*, and *PGA3*), zymogenic/chief cells (*PGA5*, *PGC*, and *LIPF*), parietal/oxyntic cells (*ATP4A*, *ATP4B*, and *CKB*), as well as endocrine cells (*CHGA*, *MS4A8*, and *SCG3*).

The remaining two clusters highly expressed *FABP5*, *NME1*, and *MYC*, but exhibited no clear cell cluster-specific gene expression patterns compared with other differentiated cell types, although one of them was mitotically active (*MKI67* and *TOP2A* double positive). Both UMAP and diffusion map indicated that these two clusters of cells were located in the middle between the surface direction and the neck/base direction, and RNA velocity analysis inferred a clear differentiation route of these cells into other differentiated cell types (Angerer et al., 2016; La Manno et al., 2018; Becht et al., 2019) (Fig. 1C and 1E). In addition, based on signaling entropy rate (SR) measurement from the package SCENT (Teschendorff and Enver, 2017), these two clusters possessed the highest SR score, indicating their high differentiation potency (Fig. 1F). Hence, we termed these two clusters as “progenitor cluster” and “cycling progenitor cluster,” and inferred them as potential gastric stem/progenitor cells. This result indicated that the differentiation of gastric cells tends to be a continuous process of gaining maturation features in a stepwise manner.

In a recent study, two distinct stem/progenitor populations in mouse gastric corpus were identified using lineage-tracing assays: one is in the base region with slow-cycling feature, and the other one is in the pit–isthmus–neck region with actively cycling feature (Han et al., 2019). And as shown in another study, mucous neck cells do not contribute substantially to the generation of zymogenic/chief cells during homeostasis, and zymogenic/chief cells maintain their own census, likely through infrequent self-replication (Burclaff et al., 2020). Thus, we checked the expression patterns of marker genes of the base stem cells, *LGR5* and *TROY*. Considering the quiescent feature of the base stem cells during homeostasis, we only detected sparse expression of these two genes in both 10× and STRT datasets of human gastric corpus, which was consistent with the results of mouse counterpart (Fig. S1E). However, due to the quiescent feature of these cells during homeostasis, we mainly focused on the progenitor cells identified above.

### The identified potential progenitor population resides in the isthmus of human gastric corpus

**Figure 2.**
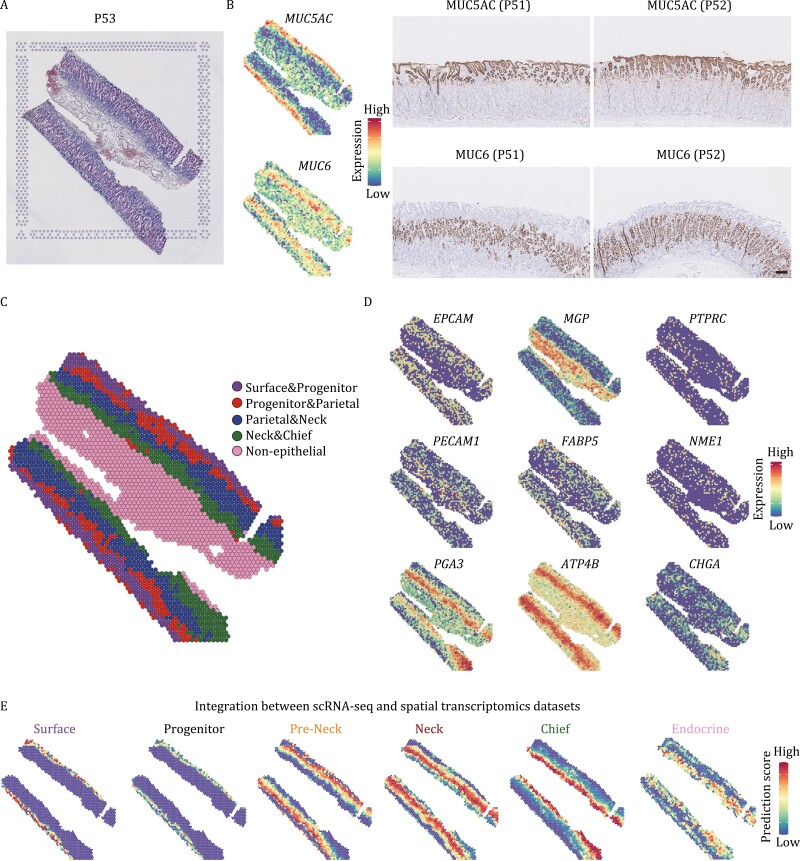
Spatial transcriptomics of human gastric corpus epithelium. (A) H&E staining of human gastric corpus section used for spatial transcriptomics. (B) Validation of spatial transcriptomics by comparison of the expression pattern of *MUC5AC* and *MUC6* in spots and immunohistochemistry staining pattern of MUC5AC and MUC6. The color key from blue to red indicates low to high expression levels, respectively. (C) Clustering result of spatial transcriptomics. Clusters are indicated by colors. (D) The expression patterns of representative marker genes in spots. (E) Integration result by transferring the scRNA-seq dataset to the spatial transcriptomics dataset.

To determine spatial distributions of the cell types identified by scRNA-seq, we performed 10× Genomics Visium experiments on two gastric corpus sections from two patients (P53 and P54) (Figs. 2A and S2A). In total, 2,323 and 1,581 spots were obtained across the sections of P53 and P54, respectively. To verify the accuracy of spatial transcriptomics, we performed immunohistochemistry staining of MUC5AC and MUC6, the marker genes of surface mucous cells and mucous neck cells, respectively. The staining results and the expression patterns of *MUC5AC* and *MUC6* in these two spatial transcriptomics datasets are quite consistent, indicating the reliability of spatial transcriptomics (Figs. 2B and S2B).

Seurat graph-based clustering analysis revealed a clear hierarchical structure in the section, and identified four epithelial and one non-epithelial clusters (Figs. 2C and S2C). According to the expression patterns of representative markers (Figs. 2D and S2D), we annotated these clusters as: Surface & Progenitor cluster; Progenitor & Parietal cluster; Parietal & Neck cluster; Neck & Chief cluster, and Non-Epithelial cluster. The expression patterns of *MUC5AC*, *MUC6*, and *PGA3* were consistent with their represented cell types, namely the surface mucous cells, mucous neck cells, and zymogenic/chief cells, respectively. The expression levels of *FABP5* and *NME1* were higher near the isthmus of the corpus glands, while the parietal/oxyntic cells (*ATP4B*) and endocrine cells (*CHGA*) were distributed in a more scattered manner along the corpus glands.

Next, we applied the “anchor”-based integration workflow of Seurat to transfer the scRNA-seq dataset to the spatial transcriptomics dataset (Hao et al., 2021). As expected, the spatial distributions of the surface mucous cells, mucous neck cells, zymogenic/chief, and endocrine cells were consistent with the previously reported results (Fig. 2E). Importantly, we found that the progenitor cluster was mapped to the isthmus of human gastric corpus, where the progenitor cells usually reside (Hoffmann, 2015), further suggesting these cells as potential stem/progenitor cells.

### Activation of WNT and EGF signaling pathways in progenitor population

According to the *in vitro* culture medium of human gastric organoids, several growth factors are indispensable to maintain the self-renewal and proliferative capacity of the stem/progenitor cells in organoids, such as EGF, WNT, R-spondin, Noggin, and A83 (inhibitor of TGF-beta signaling pathway) (Bartfeld et al., 2015). Vice versa, these related signaling pathways also tend to play important roles in the *in vivo* homeostasis of gastric stem/progenitor cells. Next, we explored the expression patterns of the related signaling pathways in our scRNA-seq and spatial transcriptomics datasets, and checked which cell population was regulated by them.

**Figure 3.**
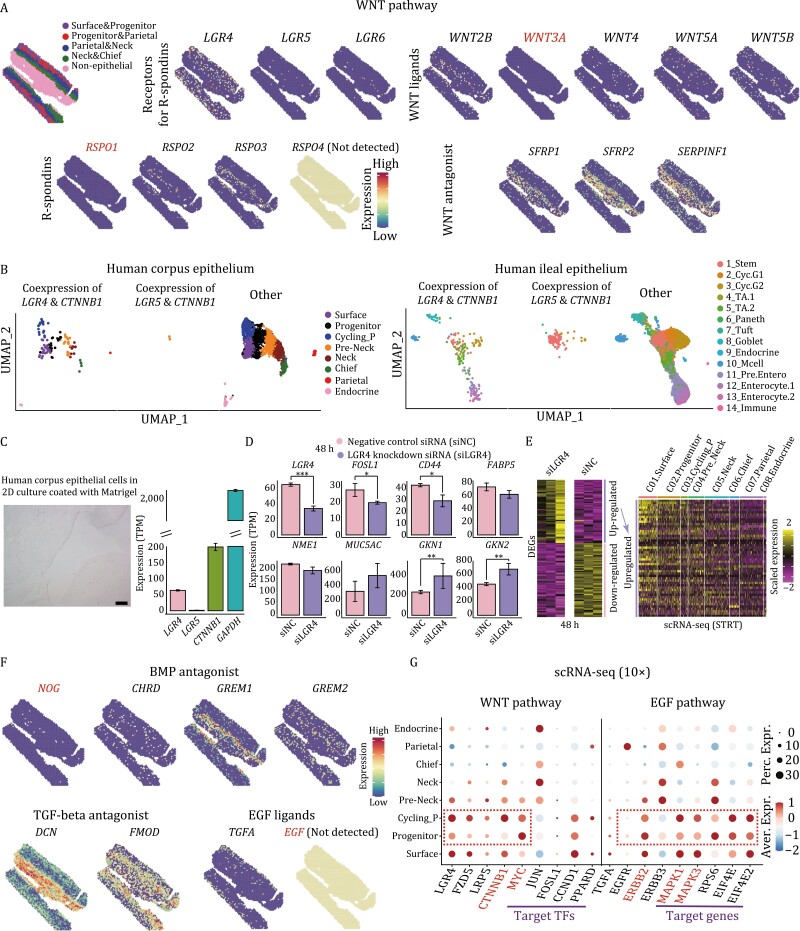
Signaling pathways crucial for the homeostasis of human gastric corpus epithelium. (A) The expression patterns of WNT signaling pathway related genes in spots. The color key from blue to red indicates low to high expression levels, respectively. (B) UMAP plots exhibiting the cells coexpressing *LGR4* & *CTNNB1* and *LGR5* & *CTNNB1* in human gastric corpus epithelium (left) and human ileal epithelium (right). Cell types are indicated by colors. TA: transit-amplifying; Mcell: microfold cell. (C) Human corpus epithelial cells in 2D culture coated with Matrigel and their gene expression levels of *LGR4*, *LGR5*, and *CTNNB1*. (D) The expression levels of representative genes in *LGR4* knockdown human corpus epithelial cells compared with those in negative control siRNA transfected ones (48 h after transfection). Significance was determined by *t*-tests. *P-value <0.05, ***P-value <0.001. (E) Heatmap exhibiting the DEGs of *LGR4* knockdown group compared with negative control siRNA transfected group (48 h after transfection) (left). On the right is the heatmap exhibiting the upregulated DEGs in the STRT scRNA-seq dataset. The color key from purple to yellow indicates low to high expression levels, respectively. (F) The gene expression patterns of BMP, TGF-beta, and EGF signaling pathways related genes in spots. (G) Dotplot exhibiting the expression pattern of WNT and EGF signaling pathways related genes in gastric corpus 10× dataset. The color key from blue to red indicates low to high expression levels, respectively. The circle size indicates the percentage of cells expressing a certain gene.

As shown in Fig. 3A, although WNT3A and RSPO1 proteins were added to the human gastric organoid medium (Bartfeld et al., 2015), they were barely expressed *in vivo*, instead, *WNT2B*, *WNT4*, *WNT5A*, and *WNT5B*, and *RSPO2* and *RSPO3*, respectively, were actually the expressed WNT ligands and R-spondins *in vivo*. Importantly, we also detected high expression levels of several WNT antagonists in epithelium and muscle layers, such as *SFRP1*, *SFRP2*, and *SERPINF1*, which might contribute to the spatially restricted activity of WNT signaling pathway *in vivo*.

*LGR5* was reported as an important player for intestine stem cells and gastric pylorus stem cells. However, in human gastric corpus epithelium, *LGR5* was barely expressed, and there were only two cells in the scRNA-seq dataset expressing both *LGR5* and *CTNNB1* (beta-catenin, the key downstream component of the canonical WNT signaling pathway) (Fig. 3B). In contrast, *LGR4* tended to be the receptor of R-spondins in human gastric corpus epithelium. The high expression levels of target transcription factors (TFs) of WNT signaling pathway, such as *MYC*, *FOSL1*, and *CCND1*, indicated that WNT signaling pathway was activated in these two progenitor cell clusters (Fig. 3G). As control, we also performed scRNA-seq for human ileal epithelial cells sampled from another two patients, and checked the expression patterns of *LGR4* and *LGR5*. We identified 19 clusters and covered all of the known cell types, namely stem/progenitor cells, Paneth cells, Tuft cells, Goblet cells, enteroendocrine cells, microfold (M) cells, and enterocytes (Fig. S3A). The DEGs and the marker genes all supported the accuracy of clustering (Fig. S3B and S3C). As expected, the coexpression of *LGR5* and *CTNNB1* was mainly enriched in the ileal stem/progenitor cells, while the coexpression patterns of *LGR4* and *CTNNB1* were scattered across all cell types (Fig. 3B). Thus, although *LGR5* is important for human ileal epithelial stem/progenitor cells, it is *LGR4* that potentially plays a critical role in human corpus epithelial stem/progenitor cells.

To validate the importance of *LGR4* in human corpus epithelium, we next performed knockdown experiments of *LGR4* in gastric stem cell lines established from normal human corpus epithelium (Fig. 3C). We digested human corpus gastric glands into single-cell suspension and then cultured them in plates coated with Matrigel matrix (see Methods). By doing so, gastric epithelial cells would expand in a monolayer format, and after several passages, cells were harvested for performing knockdown experiments. As shown in Fig. 3C, the cultured corpus epithelial cells expressed *LGR4* while barely expressed *LGR5*, which was consistent with the scRNA-seq datasets. Forty-eight hours after knockdown of *LGR4* in the cultured cells, WNT target genes, *FOSL1* and *CD44*, were downregulated. *CD44* is a major WNT target gene and acts as a positive regulator of the WNT receptor complex (Schmitt et al., 2015). In addition, gastric progenitor marker genes, *FABP5* and *NME1*, were also downregulated, while marker genes of surface mucous cells, *MUC5AC*, *GKN1*, and *GKN2*, were upregulated (Fig. 3D). When we mapped the upregulated genes in the scRNA-seq dataset, they showed higher expression levels in surface mucous cells compared to other cell types (Fig. 3E). This result indicated that *LGR4* plays important roles in maintaining the homeostasis of human corpus epithelium, and the knockdown of *LGR4* would drive gastric stem/progenitor cells to prematurely differentiate to surface mucous cells.

In addition, although Noggin, A83, and EGF were added in the human gastric organoid medium to inhibit BMP and TGF-beta, and activate EGF signaling pathways, respectively, these genes/molecules were barely expressed in the human corpus epithelium *in vivo* (Fig. 3F). We barely detected the expression of *NOG* in the spatial transcriptomics dataset, instead, the other three BMP antagonists, *CHRD*, *GREM1*, and *GREM2*, seemed to be responsible for the inhibition of BMP signaling pathway *in vivo*. *DCN* and *FMOD* were TGF-beta antagonists that are highly expressed in the human gastric corpus. While for the EGF signaling pathway, *EGF* was not detected in the spatial transcriptomics dataset, it was actually *TGFA* as the ligand. Since *TGFA* was highly expressed in the surface part of gastric corpus, its protein production might be restricted in the surface and isthmus part and hardly affect other distal cells. This result was also supported by scRNA-seq dataset that EGF signaling pathway was activated in these two progenitor clusters, because these cells resided in the isthmus of gastric corpus (Fig. 3G).

To sum up, (i) since the frequently used gastric organoid culture medium was adapted from intestine organoid culture medium, it could still be improved if mimicking the true *in vivo* expression patterns of gastric epithelial cells. (ii) The indispensable signaling pathways for gastric stem/progenitor cells, WNT and EGF, were highly activated in these two progenitor clusters compared to other mature cell types. So, we suggested these cells as the stem/progenitor cells of human gastric corpus epithelium. The developmental trajectory analyses by diffusion map and slingshot also supported this point of view and inferred four developmental routes (Fig. S3D) (Street et al., 2018): the first one is from stem/progenitor cells to surface cells; the second one is from stem/progenitor cells to neck cells; the third and fourth ones are from stem/progenitor cells to parietal cells and endocrine cells, respectively.

### *FABP5* and *NME1* are crucial for the homeostasis of gastric corpus stem/progenitor cells

**Figure 4.**
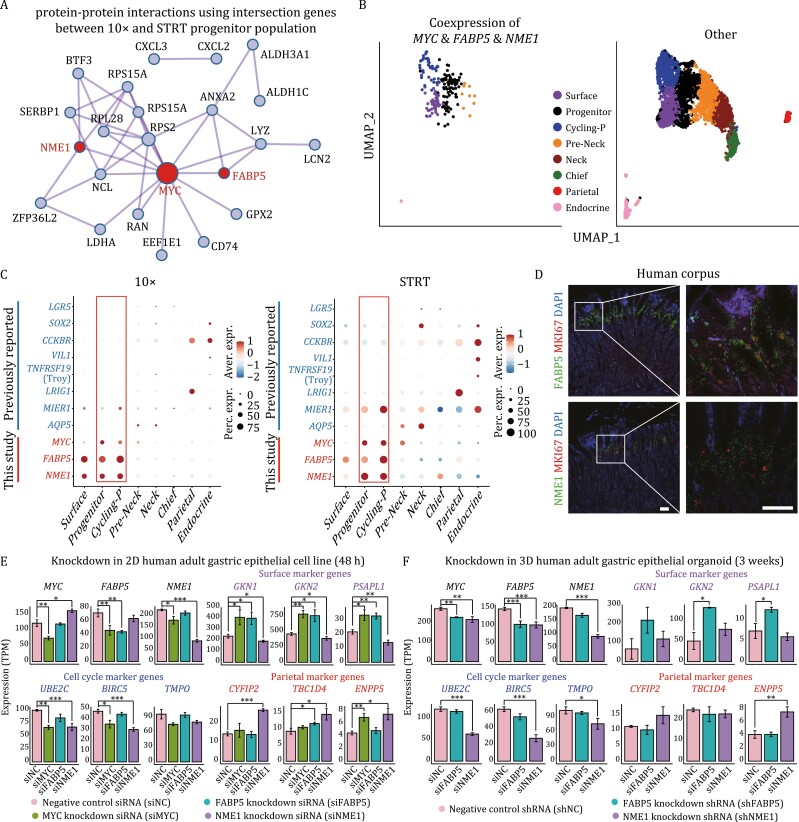
Identification of gastric corpus stem/progenitor cell marker genes. (A) Candidate stem/progenitor marker genes shown in PPI network. The network was constructed by the intersection genes between 10× and STRT progenitor population. (B) UMAP plots exhibiting the cells coexpressing *MYC* & *FABP5* & *NME1* in human gastric corpus epithelium. Cell types are indicated by colors. (C) Dotplot showing the expression patterns of the three identified genes (*MYC*, *FABP5*, and *NME1*) and previously reported marker genes of gastric stem cells in both 10× (left) and STRT (right) datasets. The color key from blue to red indicates low to high expression levels, respectively. The circle size indicates the percentage of cells expressing a certain gene. (D) RNAscope staining for *FABP5*, *NME1* and *MKI67* on human gastric corpus samples. Scale bar: 100 μm. (E) Knockdown in 2D human adult gastric epithelial cell line. Barplots exhibiting the expression levels of representative genes in *MYC*, *FABP5*, and *NME1* knockdown group compared with negative control group (48 h after transfection). Significance was determined by *t*-tests. *P-value <0.05, ***P-value <0.001. (F) Knockdown in 3D human adult gastric epithelial organoids. The expression levels of representative genes in *FABP5* and *NME1* knockdown group compared with negative control group (48 h after transfection). Significance was determined by *t*-tests. *P-value <0.05, ***P-value <0.001.

As we reasoned these two progenitor clusters as the stem/progenitor cells, to accurately identify the stem/progenitor marker genes, we took the intersection of the DEGs of progenitor clusters between 10× and STRT datasets. We visualized these genes using protein–protein interaction (PPI) network, and found that *MYC* sat in the center and interacted with most of the DEGs we identified (Fig. 4A). *FABP5* and *NME1* also interacted with *MYC* and several other DEGs. Besides, cells coexpressing *MYC*, *FABP5*, and *NME1* were highly enriched in these two progenitor clusters, thus, *MYC*, *FABP5*, and *NME1* might represent the marker genes of gastric corpus stem/progenitor cells (Fig. 4B).

Next, we compared these three identified genes with previously reported marker genes of gastric stem cells (Fig. 1C) in both 10× and STRT datasets. The 10× and STRT datasets exhibited a similar expression pattern: although lineage-tracing-based studies identified the previously reported genes as putative progenitor cells/stem markers in animal models, they were not specifically expressed in the identified human gastric progenitor clusters (Fig. 4C). *LGR5* was merely expressed in gastric epithelial cells; *LRIG1* and *AQP5* were restrictedly expressed in the parietal cells and neck cells, respectively; *CCKBR*, *TNFRSF19* (Troy), *VIL1* (villin), and *SOX2* were preferentially expressed in the endocrine cells; although *MIER1* (eR1) was highly expressed in the progenitor cells, it also showed high levels of expression in neck and endocrine cells. In contrast, *FABP5*, *NME1*, and *MYC* we identified were highly expressed in the progenitor clusters. Furthermore, we performed single-molecule RNA *in situ* hybridization assay (RNAscope) to determine the spatial expression pattern of *FABP5*, *NME1*, and a proliferation marker gene, *MKI67*, which was used as an indicator for gastric progenitor cells. Consistent with our scRNA-seq datasets, *FABP5* and *NME1* were coexpressed with *MKI67* and highly expressed in the isthmus of gastric corpus, where the stem/progenitor cells resided, further verifying their importance in the stem/progenitor cells (Fig. 4D).

To explore the roles of *MYC*, *FABP5*, and *NME1* in human corpus stem/progenitor cells, we performed the knockdown experiments of these three genes in human gastric stem cell lines and those of *FABP5* and *NME1* in human adult gastric corpus organoids. Compared with the negative control group, these three genes were successfully knocked down separately (Fig. 4E and 4F). Specifically, the knockdown of *MYC* resulted in the downregulation of *FABP5* and *NME1*, and the cell cycle-related genes, while the marker genes of surface mucous cells, such as *GKN1*, *GKN2*, and *PSAPL1*, and the marker genes of parietal/oxyntic cells, such as *ENPP5*, were upregulated (Figs. 4E and S4A). The knockdown of *FABP5* resulted in the upregulation of the marker genes of surface mucous cells, while the knockdown of *NME1* resulted in the upregulation of the marker genes of parietal/oxyntic cells. Besides, the knockdown of *NME1* could also result in the upregulation of *MYC*, and downregulation of the cell cycle-related genes. These results were also supported by the gene ontology analyses (Fig. S4B–D).

In conclusion, the knockdown of any one of *MYC*, *FABP5*, and *NME1* could break the balance and drive the premature differentiation of gastric stem/progenitor cells. The knockdown of *MYC* might drive gastric stem/progenitor cells to differentiate to surface mucous cells and parietal/oxyntic cells; while the knockdown of *FABP5* and *NME1* might mainly drive gastric stem/progenitor cells to differentiate to surface mucous cells and parietal/oxyntic cells, respectively (Fig. 4E and 4F).

### *FABP5* and *NME1* play an important role in gastric cancers

**Figure 5.**
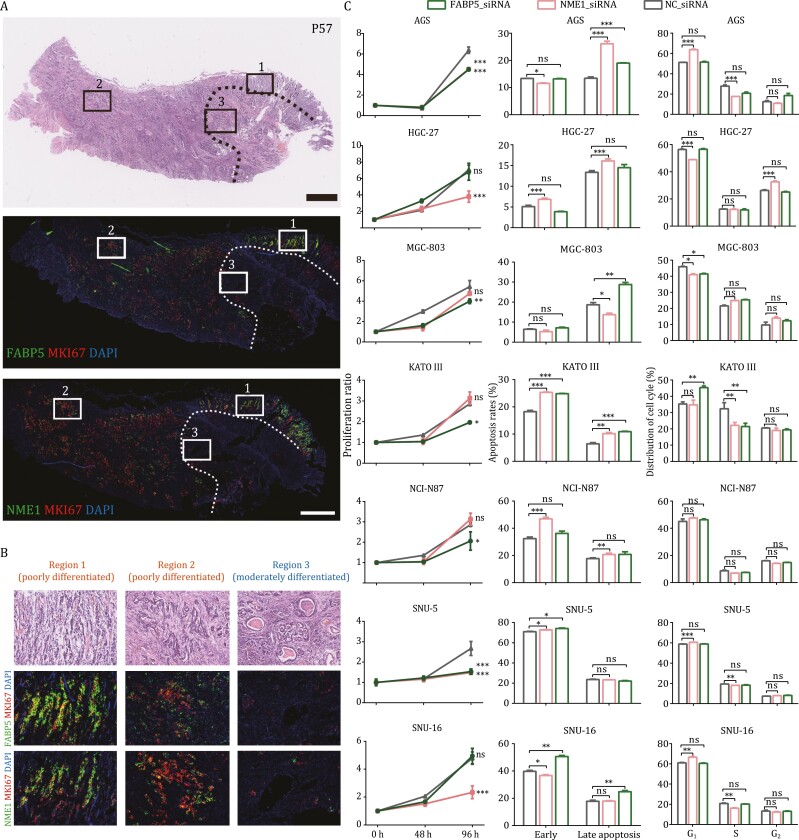
*FABP5* and *NME1* play important roles in gastric cancers. (A and B) RNAscope staining for *FABP5*, *NME1*, and *MKI67* on a gastric tumor sample. Scale bar: 1 mm. We zoomed in three regions in the tumor sample, among which, region 1 and region 2 represented two poorly differentiated regions; while region 3 exhibited duct represented a moderately differentiated region. Correspondingly, *FABP5*, *NME1*, and *MKI67* were highly expressed in region 1 and region 2, while they were barely expressed in region 3. Tumor tissues in the region circled by the dash line was more differentiated than those in other regions in the H&E staining section, where the expression levels of these three genes were also low. (C) From left to right indicate the results of cell proliferation, apoptosis, and cell cycle experiments after *FABP5* and *NME1* knockdown in seven different gastric cancer cell lines. Significance was determined by *t*-tests. *P-value < 0.05, ***P-value < 0.001.

According to mouse model-based lineage-tracing studies and human sample-based clonality analyses, gastric cancers tend to originate from tissue-resident stem cells (Hayakawa et al., 2017). Since *FABP5* and *NME1* were proven to be important for gastric epithelial stem/progenitor cells, we asked whether they also play a crucial role in gastric cancers. Thus, we performed RNAscope staining and detected a coexpression pattern of *FABP5*, *NME1*, and *MKI67* across the tumor tissues (Figs. 5A and S5A). Furthermore, we marked three regions in the tumor tissue; region 1 and region 2 represented two poorly differentiated adenocarcinoma regions with masses of tumor cells scattered in the stroma, while region 3 represented a moderately differentiated region with tumor cells forming tubular structures (Fig. 5A and 5B). Correspondingly, *FABP5*, *NME1*, and *MKI67* were highly expressed in region 1 and region 2, while they were much more sparsely expressed in region 3. These results indicated that higher expression levels of these three markers were mainly detected in the more poorly differentiated adenocarcinoma regions, while the expression levels of them in the more differentiated adenocarcinoma regions in the same tumor section were much lower.

This pattern was similar to that of normal gastric epithelium, where most cycling epithelial cells were also progenitor cells. Since cycling and stem-like malignant cells might possess greater tumor-propagating potential, we wonder whether the destruction of their regulatory network would reduce the propagation of gastric cancers. Thus, to test this hypothesis, we knocked down *FABP5* and *NME1* in seven different gastric cancer cell lines, including AGS, HGC-27, MGC-803, KATO III, NCI-N87, SNU-5, and SNU-16 (Figs. 5C and S5B–D). In all seven gastric cancer cell lines, either or both of *FABP5* and *NME1* knockdown would result in a significant reduction of proliferation, an increase of apoptosis. Besides, the cell cycle states of all cell lines except NCI-N97 were also affected by either or both of *FABP5* and *NME1* knockdown. In conclusion, *FABP5* and *NME1* were not only identified as normal gastric corpus stem/progenitor markers, but also proven to be important for the propagation in gastric cancer cells.

### Chromatin accessibility landscapes of gastric corpus epithelium

**Figure 6.**
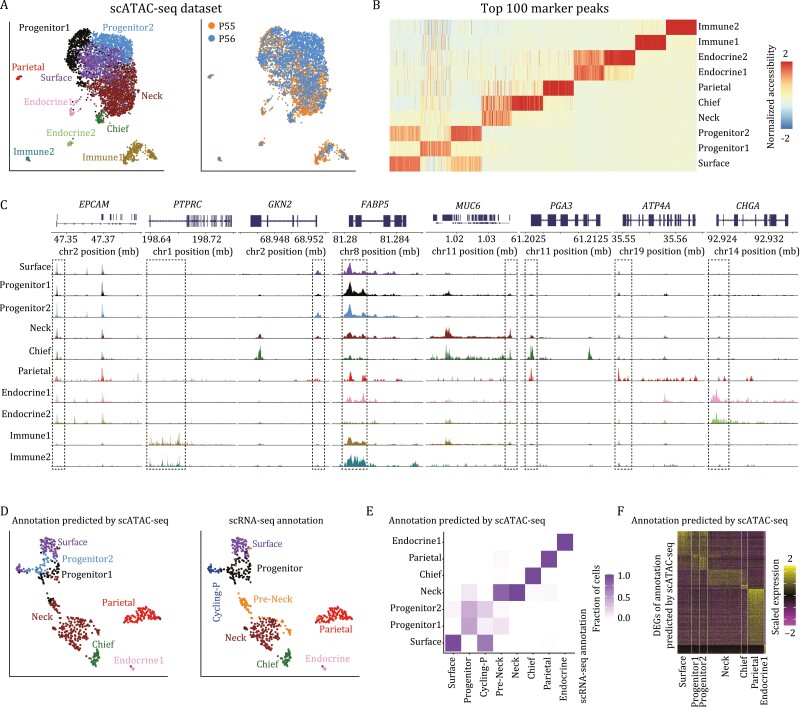
Chromatin accessibility landscapes of human gastric corpus epithelium. (A) UMAP plots exhibiting the identified clusters (left) and sample information (right) of scATAC-seq dataset in human gastric corpus. Clusters and samples are indicated by colors. (B) Heatmap exhibiting the top 100 marker peaks of each cluster. The color key from blue to red indicates low to high normalized accessibility, respectively. (C) Genomic tracks exhibiting the accessibility of representative marker genes in aggregated scATAC-seq clusters. (D) UMAP plots exhibiting CCA integration result between gene activity scores of scATAC-seq and gene expression levels of scRNA-seq. The left panel is the annotation predicated by scATAC-seq, and the right panel is the original scRNA-seq clustering result. Clusters are indicated by colors. (E) Heatmap exhibiting the comparison between the annotation predicated by scATAC-seq and the original scRNA-seq clustering result. The color key from white to purple indicates low to high fraction of cells, respectively. (F) Heatmap exhibiting the DEGs of scRNA-seq dataset annotated by scATAC-seq. The color key from purple to yellow indicates low to high expression levels, respectively.

Although we have identified the potential stem/progenitor cells in gastric corpus epithelium, the epigenetic regulatory mechanisms maintaining their homeostasis and differentiation remain elusive. Due to the technical limitations of scRNA-seq in detecting low-abundance transcripts, such as TFs, we performed 10× Genomics scATAC-seq to infer the regulatory network of human gastric corpus epithelium using two patient samples. After stringent quality control, we obtained 5,076 cells with a median of 53,039 fragments per cell. Next, we performed the downstream analysis with Signac (Hao et al., 2021) and batch correction with Harmony (Korsunsky et al., 2019), and obtained 10 clusters of cells with distinct chromatin accessibility patterns (Figs. 6A, 6B, and S6). Based on the accessibility of selected marker genes identified by scRNA-seq dataset, we annotated these 10 clusters as surface mucous cells, two clusters of progenitor cells, mucous neck cells, zymogenic/chief cells, parietal/oxyntic cells, two clusters of endocrine cells, as well as two clusters of immune cells (Fig. 6C).

The epithelial clusters of scATAC-seq were nicely matched with those from the scRNA-seq result except that there was no distinct mitotic cluster, which is consistent with previous reports that the chromatin accessibility landscapes of cells at different cell cycle phases were of high similarities to each other (Hsiung et al., 2015). In addition, the canonical correlation analysis (CCA) integration result between gene activity scores of scATAC-seq and gene expression levels of scRNA-seq was also consistent across both modalities (Fig. 6D and 6E). The expression pattern of scRNA-seq dataset annotated by scATAC-seq dataset was also consistent with the above result in Fig. 1D, and each cluster had specific DEGs, indicating the accuracy of CCA integration result (Fig. 6F).

### Epigenetic regulatory network of human gastric corpus epithelium

**Figure 7.**
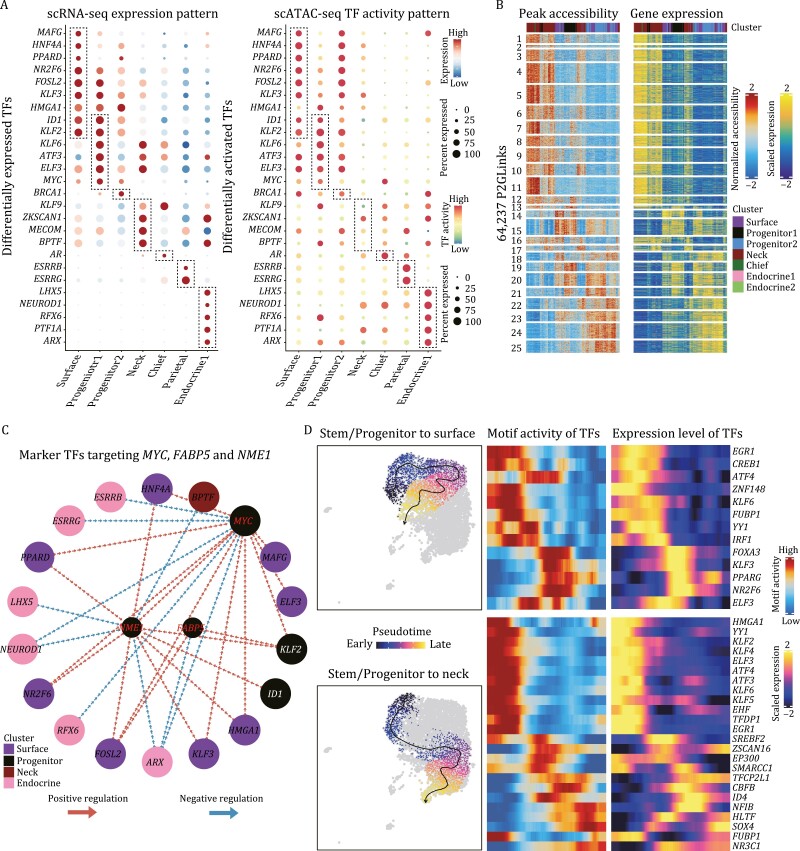
Epigenetic regulatory network of human gastric corpus epithelium. (A) Dotplots exhibiting the differentially expressed TFs (left) and differentially activated TFs (right) in scRNA-seq and scATAC-seq datasets, respectively. The color key from blue/green to red indicates low to high expression levels, respectively. The circle size indicates the percentage of cells for a certain TF. (B) Heatmaps exhibiting the peak accessibility (left) and expression levels (right) of 64,237 peak-to-gene pairs. The color key from blue to red/yellow indicates low to high normalized accessibility/expression levels, respectively. (C) Regulatory network of cell type specific TFs targeting the three stem/progenitor marker genes, *MYC*, *FABP5*, and *NME1*. Red and blue arrows indicate positive and negative regulation, respectively. (D) Differentiation routes of corpus stem/progenitor cells to surface mucous cells (up) and neck mucous cells (bottom). On the right are the heatmaps exhibiting the TF motif activity and expression levels along the differentiation routes. The color key from blue to red/yellow indicates low to high motif activity/expression levels, respectively.

To assess the epigenetic regulatory programs of gastric corpus epithelium, we used chromVAR (Schep et al., 2017) to infer TF-associated chromatin accessibility in different epithelial clusters, and found that each cluster could be defined by specific TF activity patterns (Fig. S7A). To reduce the false-positive rate, we performed a stringent analysis by taking the intersections between differentially active TFs identified by scATAC-seq and differentially expressed TFs identified by scRNA-seq. As shown in Fig. 7A, six TFs, namely *ID1*, *KLF2*, *KLF6*, *ATF3*, *ELF3*, and *MYC*, were found to be specifically expressed and activated in the progenitor 1 cluster. Meanwhile, we also identified several marker TFs in other cell types, such as *MAFG*, *HNF4A*, *PPARD* for surface mucous cells; *KLF9*, *ZKSCAN1*, *MECOM*, *BPTF* for mucous neck cells; *AR* for chief cells; *SERRB*, *ESRRG* for parietal cells; and *LHX5*, *NEUROD1*, *RFX6*, *PTF1A*, *ARX* for endocrine cells. These identified critical TFs were not only highly expressed but also specifically activated in certain cell types, and thus were thought to play crucial roles for these types of cells.

After we have identified the cell type-specific TFs in human corpus epithelium, we asked how they regulate target genes to maintain cellular homeostasis and differentiation. To explore the direct downstream targets of the identified TFs, we first identified the “peak-to-gene links” in ArchR, which seeks the correlations between peak accessibility and gene expression based on the CCA integration result between scATAC-seq and scRNA-seq datasets. In total, we identified 64,237 peak-to-gene pairs in the corpus epithelium (Fig. 7B; Table S4). For each peak-to-gene pair, if the peak contained a certain binding motif of a TF, the linked gene was considered the potential target of the corresponding TF. As shown in Fig. S7B, most of the cell type-specific DEGs were inferred as the direct downstream targets of the TFs and thus could be regulated by these TFs, indicating the potential involvement of these TFs in the homeostasis of human corpus epithelium. Take the TF, *MYC* as an example, we regarded the up- and downregulated genes identified in the knockdown experiments as validated *MYC*-regulated genes, and many of them were not only the potential target genes of *MYC* but also the corpus DEGs (Fig. S7C). This result indicated that *MYC* could activate some of its target genes whereas repress other target genes, and probably both are important for the maintenance of the homeostasis of human corpus epithelium. Moreover, the repressed target genes of *MYC* mainly belonged to surface mucous cells and parietal/oxyntic cells, and were upregulated after the knockdown of *MYC*, indicating the premature differentiation toward these two directions after *MYC* was knocked down, as also shown in Fig. 4E.

Among these cell type-specific TFs, several of them could potentially target the three stem/progenitor marker genes, *MYC*, *FABP5*, and *NME1* (Fig. 7C). Intriguingly, we also found that surface mucous cell and progenitor cell-specific TFs tend to positively regulate *MYC*, *FABP5*, and *NME1*, while mucous neck cell and endocrine cell-specific TFs, on the other hand, tend to negatively regulate them. Finally, we performed the developmental trajectory analysis in ArchR to reveal the transient changes in TFs along the two main differentiation directions: from stem/progenitor to surface mucous cells and to neck mucous cells (Fig. 7D). We identified 13 and 24 TFs exhibiting transient changes in both motif activities and gene expression levels for these two differentiation routes, respectively.

## Discussion

Human stomach is not only important for storing and digesting food, but can also defend against food-borne microbes through the secretion of gastric acid (Hunt et al., 2015). There are thousands of gastric units in the human gastric mucosa, and each unit contains several different cell types on the surface and in the gastric glands. Among these cell types, gastric epithelial stem/progenitor cells are important for stomach homeostasis, and the dyshomeostasis of human stomach will contribute to tumor formation. However, cellular and molecular knowledge of human stomach remains limited. Recently, Clevers group performed scRNA-seq analysis to study the human upper gastrointestinal organs, including the esophagus, stomach, and duodenum (Busslinger et al., 2021). For the stomach, they described the cellular composition of human stomach, and then mainly focused on the comparisons between human and mouse stomachs to reveal the species-specific expression differences, while lacking deep exploration and discussion of human gastric stem/progenitor cells. Although the identity of human gastric stem/progenitor cells is still controversial, a few efforts have been done to characterize their molecular features. In this study, we combined scRNA-seq, scATAC-seq and spatial transcriptomics techniques to systematically analyze human gastric corpus epithelium, aiming to identify the gastric stem/progenitor cells, characterize their molecular markers and reveal the gene-regulatory networks that are important for gastric homeostasis.

We identified a stem/progenitor population in the isthmus of human gastric corpus. These stem/progenitor cells exhibited relatively higher expression levels of *FABP5*, *NME1*, *MYC*, and proliferation marker genes, and higher differentiation potency, but no signs of differentiation or maturation compared with other differentiated cell types (Fig. 1D and 1F). The spatial transcriptomics results and smFISH (RNAscope) staining also mapped the stem/progenitor cells to the isthmus of human gastric corpus, where the progenitor cells usually reside (Figs. 2E and 4D). Furthermore, the indispensable signaling pathways (WNT and EGF) for gastric stem/progenitor cells were highly activated in these stem/progenitor cells (Fig. 3G). Besides, we also found that *LGR4*, but not *LGR5*, was responsible for the activation of WNT signaling pathway in human corpus epithelial stem/progenitor cells. In mouse gastric corpus, two distinct stem/progenitor populations have been identified: one is in the base region with a slow-cycling feature, and the other one is in the pit–isthmus–neck region with an actively cycling feature (Han et al., 2019). Indeed, we also detected sparse expression of marker genes of the base stem cells, *LGR5* and *TROY*, in human gastric corpus. However, due to the ethical limit in human with genetic labeling and the lack of proliferating features in these cells during homeostasis, scRNA-seq methods with higher resolution and better *in vitro* models of human gastric corpus will be needed to comprehensively characterize these cells.

We found that human gastric corpus stem/progenitor cells highly expressed *FABP5* and *NME1*, but did not specifically express previously reported stem cell markers identified by lineage-tracing-based studies in mice, such as *LGR5*, *LRIG1*, *AQP5*, *CCKBR*, *Troy*, *villin*, *SOX2*, and *eR1* (Fig. 4C). These differences may be ascribed to two reasons. First, these gastric stem cell markers were mainly identified by mouse models, and there may exist species differences between human stomach and murine one (Saenz and Mills, 2018; Busslinger et al., 2021). Second, these marker genes may be also due to lineage-tracing problem, because several studies have reported the stem cell-like activity in multiple promoters of reporter genes upon lineage tracing (Kretzschmar and Watt, 2012; Hayakawa et al., 2015b; Li et al., 2016). In addition, the identified gastric stem/progenitor marker genes here, that is, *FABP5* and *NME1*, are also important for the propagation in gastric cancers as proved by *in vivo* gastric cancer samples and several gastric cancer cell lines (Fig. 5).

Last but not least, we also revealed the gene-regulatory network of human gastric corpus epithelium. We performed scATAC-seq analysis to explore the epigenetic regulatory mechanisms maintaining gastric homeostasis and differentiation, which could complement the technical limitations of scRNA-seq in detecting TFs. We identified six TFs that are specifically expressed and activated in the gastric stem/progenitor cells, and several marker TFs in other cell types (Fig. 7A). Further functional experiments are needed to reveal their roles for certain cell types.

In summary, to our knowledge, this is the first work to systematically study human gastric corpus epithelium at single-cell multi-omics levels, where we uncover the spatially resolved expression landscape and dynamic gene-regulatory networks of human gastric corpus epithelium. Our work paves the way for understanding the cellular diversity and homeostasis of human gastric corpus epithelium, and has the potential to inspire novel strategies for the treatment of gastric diseases including gastric cancers.

## Methods

### Human gastric corpus and ileal specimen sampling

This research was approved by the Ethics Committee of Peking University Third Hospital (License No. IRB00006761-M2016170), and all patients signed written informed consent for this study. Gastric corpus samples were collected immediately after surgical resection. Single gland cells were isolated using a previously described protocol (Bartfeld et al., 2015). Briefly, the tissues were washed for three times with cold chelation buffer and cut into 5-mm pieces. These pieces were transferred into a cold chelation buffer with EDTA followed by incubation for 30 min to 1 h at 4°C. Then, the pieces were placed on 10-cm dishes, and glass slides were put on top of them to make the glands visible in solution. Finally, glands were collected by centrifugation and resuspended with TrypLE at 37°C for 5 min to dissociate them into single-cell suspension. Ileal crypt samples were collected from two right-sided colon cancer patients immediately after surgical resection, and then were dissociated into single-cell suspension.

### Human corpus organoid culture (3D) and monolayer epithelial cell culture (2D)

Gland cells were sampled from normal gastric corpus tissues using a previously described protocol with some modifications (Bartfeld et al., 2015). Briefly, the tissue was washed and cut into small pieces after removing fat and mucus. Tissue fragments were incubated in 1× Ca^2+^/Mg^2+^-free DPBS supplemented with 10 mmol/L EDTA and 0.5 mmol/L DTT, shaking on ice for 1 h. Glands were isolated by applying pressure with a glass slide, and were collected with Advanced DMEM/F12 and filtered through 100-μm filter. For organoid culture, corpus glands were embedded into Matrigel on 24-well plate, 50 μL per Matrigel bead. For monolayer cell culture, corpus glands were dissociated into single-cell suspension with TrypLE for 3–5 min at 37°C on a thermomixer, and were then filtered through 40-μm filter. Cells were plated on Matrigel-coated plates which required complete air drying before use. Culture medium contained Advanced DMEM/F12, 50% L-WRN-conditioned medium, 10 mmol/L HEPES, 1× GlutaMAX, 1× penicillin/streptomycin, 1× B27, 1× N2, 100 ug/mL primocin, 200 ng/mL FGF10, 50 ng/mL EGF, 1 nmol/L gastrin, 2 μmol/L A83-01, 10 mmol/L Nicotinamide, 1 mmol/L N-Acetylcysteine, and 10 μmol/L Y27632. Medium was changed every 3–4 days.

### Cell-line culture

All gastric cancer cell lines were purchased from ATCC. The AGS cell line was maintained in F-12K medium containing 10% FBS and 100 units/mL penicillin and 100 μg/mL streptomycin. The growth conditions of MGC-803, SNU-16, and NCI-N87 were RPMI-1640 medium with 10% heat-inactivated FBS, and the HGC-27 was were routinely cultured in RPMI-1640 medium and supplemented with 20% FBS. The KATO III was grown in Iscove’s Modified Dulbecco’s Medium (IMDM) with 20% FBS. The SNU-5 was cultured using IMDM with 20% FBS. All the cells above were incubated at 37°C in a 5% CO_2_ humidified atmosphere.

### Transfection of siRNA and shRNA

Gastric epithelial cells were dissociated with TrypLE for 8–10 min at 37°C, then two volumes of Advanced DMEM/F12 supplement with B27 and N2 were added. Cells were centrifuged and resuspended in the culture medium. Transfection of siRNA was performed in the cell suspension of 6 × 10^4^ cells per well using siRNAsuper reagent (IGE Biotech), and plated on Matrigel-coated 48-well plate. Cells were collected for bulk RNA-seq analysis after 48 h of transfection. For gastric cancer cell lines, siRNA was transfected into cells using Lipofectamine RNAiMAX reagent (Thermo Fisher Scientific) when cells had reached 50%–70% confluence. The siRNA was synthesized by Sangon Biotech (Shanghai, China), and detailed sequence was listed in the table below:

FABP5:5ʹ-CACCUGUACUCGGAUCUAUTT-3ʹ,5ʹ-AUAGAUCCGAGUACAGGUGTT-3ʹ; NME1:5ʹ-CCCUGAGGAACUGGUAGAUTT-3ʹ,5ʹ-AUCUACCAGUUCCUCAGGGTT-3ʹ; MYC:5ʹ-AGACCUUCAUCAAAAACAUUU-3ʹ, 5ʹ-AUGUUUUUGAUGAAGGUCUUU; LGR4:5ʹ-GAAAGUAAACUGUGGUCAAUU-3ʹ,5ʹ-UUGACCACAGUUUACUUUCUU-3ʹ; NC:5ʹ-UUCUCCGAACGUGUCACGUTT-3ʹ,5ʹ-ACGUGACACGUUCGGAGAATT-3ʹ

For transfection of shRNA, corpus organoids were dissociated into single cells with TrypLE for 8–10 min at 37°C, then filtered through 40-μm filter and counted. Transduction of shRNA-FABP5, shRNA-NME1, and shRNA-NC lentivirus were performed in cell suspension on 24-well plate, respectively, using 20,000 cells in 500 μL culture medium supplemented with Y27632. Cells were slowly rocked on a shaker in the incubator at 37°C for 6 h to avoid attachment and allow efficient transduction of lentivirus. Cells were collected for bulk RNA-seq analysis after 3 weeks of transfection. The shRNA lentivirus was packaged by Hanbio Biotechnology (Shanghai, China), using the same target sequence as siRNA.

### H&E and immunohistochemistry staining

Tissues were fixed in 10% neutral formalin overnight, dehydrated through graded ethanol, embedded in paraffin, and processed using standard methods. Paraffin-embedded tissue blocks were sliced into 5-μm-thick sections on a microtome. H&E staining was performed on these 5-μm-thick slides. For immunohistochemistry staining, slides were deparaffinized and hydrated before endogenous peroxidase activity was blocked. The slides were boiled for antigen retrieval in pH 6.0 citrate buffer and blocked by 4% BSA. We then applied appropriately diluted primary antibodies to the slides and incubated them in a humidified chamber at 37°C for 2 h. The following primary antibodies were used: anti-MUC5AC antibody (Abcam, ab3649), anti-MUC6 antibody (Abcam, ab212648), anti-gastrin antibody (ThermoFisher Scientific, PA5-32422), anti-KI67 antibody (Abcam, ab15580), anti-FABP5 antibody (Santa Cruz, sc-365236), and anti-CDNK1A antibody (ThermoFisher Scientific, MA5-14949). Samples were incubated with HRP-conjugated secondary antibodies for 30 min. We applied 50 μL DAB to the slides to reveal the color and allowed the color to develop for <5 min under microscopy until the desired color intensity was reached. We counterstained the slides by immersing them in hematoxylin. Images were captured with a Nikon Eclipse 90i and were reviewed by two independent pathologists.

### Single-molecule RNA fluorescence *in situ* hybridization (smFISH)

Fresh tissues were first fixed in 4% paraformaldehyde (PFA) in 1× PBS for 24 h at 4°C. The fixed samples were washed with 1× PBS, followed by immersion in 20% sucrose in 1× PBS and 30% sucrose in 1× PBS at 4°C before being soaked in OCT cryo-embedding medium and frozen in a dry ice bath. Fixed-frozen tissues were then cut into 15-μm-thick sections and placed on microscope glass slides for subsequent smFISH processing. smFISH was performed using RNAscope Multiplex Fluorescent Reagent Kit v2 and RNAscope probes Hs-NME1 (470651), Hs-FABP5-C3 (566111-C3), Hs-MKI67-C2 (591771-C2) (Advanced Cell Diagnostics, ACD) according to the manufacturer’s protocols. The slides were stained with DAPI before being mounted with KPL-mounting medium. Images of tissue sections were taken with a Nikon A1R confocal microscope.

### Cell proliferation assay, cell apoptosis analysis, and cell cycle analysis

For cell proliferation assay, cells were harvested and plated in 12-well plates at 10,000–20,000 cells per well in culture medium after 24 h of transfection. The cells were counted after 48 h and 96 h of culture. For gastric organoids, transduction of shRNA-FABP5, shRNA-NME1, and shRNA-NC lentivirus were performed in cell suspension on 24-well plate, respectively, using 20,000 cells in 500 μL culture medium supplemented with Y27632. Cells were slowly rocked on a shaker in the incubator at 37°C for 6 h to avoid attachment and allow efficient transduction of lentivirus. Then cells were collected and centrifuged, resuspended in Matrigel, and seeded into 96-well plate, 5,000 cells in 10 μL Matrigel bead per well. Cell viability was assayed using CellCounting-Lite 2.0 Luminescent Cell Viability Assay (Vazyme, DD1101) on day 0, day 2, and day 7.

For cell cycle analysis, cells were fixed by adding prechilled 70% ethanol to the cell suspension overnight, then cells were resuspended by FxCycle™ PI/RNase staining solution (Thermo Fisher Scientific) for 30 min at room temperature after washing by PBS. For cell apoptosis analysis (Apoptosis Kit, Invitrogen), cells were washed with cold PBS and resuspended by 100 μL 1× annexin-binding buffer, and add 1 μL PI working solution (100 μg/mL) and 5 µL Alexa Fluor® 488 annexin V into cell suspension, then incubate the cells at room temperature for 15 min. After the incubation period, add 400 μL of 1× annexin-binding buffer, mix gently, and keep the samples on ice. Then, we analyzed the results by flow cytometry using BD LSRFortessa^TM^ cytometry within 1 h.

### RNA isolation and RT-qPCR

RNA from the cells was extracted using the RNeasy Mini Kit (QIAGEN, 74106) following the manufacturer’s instructions. Extracted RNA was reverse transcribed into cDNA using the HiScript III RT SuperMix for qPCR kit (Vazyme, R323). qPCR samples were amplified with NovoStart SYBR qPCR SuperMix Plus (Novoprotein, E096) in 20 μL of total volume.

### scRNA-seq, scATAC-seq, and spatial transcriptomics library construction

In order to balance the throughput and accuracy, we used two mature single-cell RNA-seq techniques: 10× and STRT. Our STRT method was modified from the original STRT protocol and has been described in previous studies (Islam et al., 2011; Li et al., 2017; Dong et al., 2018). The barcode information was described in Table S3. Briefly, first-strand cDNA was generated by a reverse-transcription reaction, followed by 18 cycles of PCR to amplify the cDNA. The amplified cDNA was pooled together before biotin tags were added to the 3ʹ end of the amplified cDNAs. We sheared the 300 ng of amplified cDNA into 300-bp fragments using a Covaris S2 system and used C1 beads to capture the 3ʹ terminal of the cDNA. The library was prepared using a KAPA Hyper Prep Kit (KAPA Biosystems).

For 10× Genomics scRNA-seq method, libraries were generated using the 10× Genomics Chromium platform and Chromium Single Cell 3ʹ Reagent Kits v2 (corpus samples) and v3 (ileal samples) following manufacturer’s instructions. All the prepared libraries were sequenced on an Illumina HiSeq 4000 platform using 150-bp paired-end sequencing. 10× Genomics scATAC-seq technique and 10× Visium spatial transcriptomics technique were performed following manufacturer’s instructions.

### Bulk mRNA-seq

Total RNA was extracted using the RNeasy Mini Kit (QIAGEN) following the manufacturer’s instructions. We performed mRNA isolation using the NEB Poly (A) mRNA Magnetic Isolation Module (NEB) and constructed an RNA library using the KAPA Hyper Prep Kit (KAPA Biosystems). The RNA library was sequenced on an Illumina HiSeq 4000 platform using 150-bp paired-end sequencing.

### Processing of scRNA-seq data

For corpus STRT dataset, barcodes and unique molecular identifiers (UMIs) were extracted from the R2 reads using UMI-tools (Smith et al., 2017). We removed the template switch oligo and polyA tail sequence from the obtained reads. Meanwhile, we also discarded reads with low-quality bases using seqtk. Subsequently, the clean reads were aligned to the human GRCh38 genomes using STAR (Dobin et al., 2013). We used featureCounts (Yang et al., 2014) to count the uniquely mapped reads and quantified the UMIs with UMI-tools. For corpus 10× dataset, we used Cell Ranger 2.2.0 with default mapping arguments to process the raw data. Reads were aligned to the human GRCh38 genome. After obtaining the UMI expression table, for STRT dataset, we removed cells with fewer than 1000 detected genes and 10,000 detected transcripts; and for 10× dataset, we removed cells with fewer than 200 detected genes. Cells with high mitochondrial gene expression fractions were also removed.

To reduce the batch effect arising from patients’ differences, we first used Harmony to correct the bath effect based on patient information (Korsunsky et al., 2019). Briefly, we used Seurat to identify highly variable genes (HVGs) using the cutoff: average expression >0.5 and dispersion greater >0.5 for STRT dataset, average expression >0.125, and dispersion greater >0.5 for 10× dataset (Satija et al., 2015). Then we used these identified HVGs to perform PCA and corrected the principal components using Harmony. Finally, these corrected principal components were used as inputs for UMAP analysis and clustering through a graph-based method in Seurat.

For human ileal scRNA-seq dataset, we used Cellranger v3.1.0 (10× Genomics) to deal with the raw reads and quantify the expression level. Next, the UMI count matrix was analyzed using Seurat pipeline. We discarded cells with gene numbers below 1000, UMI counts below 1,000, and mitochondrial percentage above 30%. For SCORE analysis, 8,000 HVGs were chosen using FindVariableFeatures (nfeatures = 8,000, selection.method = “vst”) (Dong et al., 2022). The overall dimensionality reduction and clustering were performed using all the obtained modules.

For RNA velocity analysis, velocyto v0.17.17 was used to generate the loom file containing the spliced and unspliced expression matrices information for each sample (La Manno et al., 2018). Scvelo v0.2.4 was used to estimate the RNA velocity using group DEGs (Bergen et al., 2020). The first- and second-order moments for velocity estimation were calculated by scvelo.pp.moments with n_pcs=30, n_neighbors=30. scvelo.tl.velocity with mode=’ dynamical’ was used to learn the full transcriptional dynamics of splicing kinetics.

### Processing of scATAC-seq data

We used cellranger-atac 1.2.0 with default arguments to process the scATAC raw data. “Signac” was used to integrate the two corpus samples and arrow files were created using “createArrowFiles” function with default arguments from “ArchR 0.9.5” package (Granja et al., 2021; Hao et al., 2021). The enriched motifs of marker peaks were identified by “peakAnnoEnrichment” function with threshold as “FDR <= 0.1 & Log2FC >= 0.5”. TF motif activity was calculated using “addDeviationsMatrix” function. Trajectory analysis was fulfilled by “getTrajectory” function. In order to link peak accessibility and gene expression, we identified the peak-to-gene links using “addPeak2GeneLinks” and “getPeak2GeneLinks” functions.

For the construction of TF and gene-regulatory network, we used the peak-to-gene links that satisfy the following criteria: (i) the peak contains the motif for specific TF. (ii) The target gene belongs to the “Marker Gene” of specific cluster based on the STRT data. Notably, the color of the node represents TF or specific cluster “Marker Gene,” and the interaction color represents the correlation of expression between the linked TF and gene (red: positive correlated, blue: negative correlated).

### Processing of spatial transcriptomics data

The raw data of 10× Genomics Visium data were processed by “spaceranger 1.2.1.” The downstream analysis was fulfilled by “Seurat” package (Hao et al., 2021). “LogNormalize” method from “NormalizeData” function was used to normalize the “spatial” assay, and 3000 variable features were used to do dimensionality reduction. To determine the spatial distribution of the cell types identified by scRNA-seq, “TransferData” function was used to transfer the cell clusters from scRNA to spatial clusters using anchors identified by “FindTransferAnchors” function using top 50 Endocrine DEGs for Endocrine transferring and all variable features for other cell-type transferring.

### Differentiation potency calculation

The differentiation potency for each cell was calculated using Signaling Entropy Rate (SR) measure from the package SCENT (Teschendorff and Enver, 2017). The PPI network was obtained from BioGRID (Version 3.5.168) (Chatr-Aryamontri et al., 2017).

### Differentially expressed gene analysis and enrichment analysis

For scRNA-seq, we used the FindAllMarkers function in Seurat (test.use = “wilcox”, min.pct = 0.25, logfc.threshold = 0.25) to identify DEGs for each cluster and the FindMarkers function in Seurat (test.use = “wilcox”, min.pct = 0.25, logfc.threshold = 0.25) to identify DEGs for two given clusters. For bulk RNA-seq, we performed the DEG analysis using DE-seq2 (Love et al., 2014). We performed enrichment analysis in Metascape (Tripathi et al., 2015).

## Supplementary Material

pwac059_suppl_Supplementary_Materials

pwac059_suppl_Supplementary_Table_S1

pwac059_suppl_Supplementary_Table_S2

pwac059_suppl_Supplementary_Table_S3

pwac059_suppl_Supplementary_Table_S4

## Data Availability

All sequencing data generated in this study are deposited in the Genome Sequence Archive (GSA) database (accession number: HRA002917).

## Abbreviations

CCA: canonical correlation analysis
scATAC-seq: single-cell assay for transposase accessible chromatin sequencing
scRNA-seq: single-cell RNA sequencing
SR: signaling entropy rate
TF: transcription factor

## Code availability

All data analyses were followed by standard programs and packages and code is available on request.
