# Supplementary material for: Spatially resolved expression landscape and gene-regulatory network of human gastric corpus epithelium
Source: Protein Cell. 2022 Nov 15;14(6):433–47. doi: 10.1093/procel/pwac059 (PMC10319429; doi:10.1093/procel/pwac059)
Supplement: pwac059_suppl_Supplementary_Materials [file pwac059_suppl_supplementary_materials.pdf]

Supplemental Figure legends

Fig. S1. Sampling information of human gastric corpus epithelium

(A) Scatter plots showing the distribution of UMIs and genes across all used cells. The red lines indicate the thresholds of quality control.

(B) UMAP plots exhibiting the human corpus cells of the 10X (left) and STRT (right) datasets. Cells sampled from different patients are in different colors.

(C) Heatmap showing DEGs of each cluster in gastric corpus STRT dataset. The color key from purple to yellow indicates low to high expression levels, respectively.

(D) Violin plots exhibiting the expression of the representative marker genes of gastric cell type in the 10X dataset. Different cell types are in different colors.

(E) UMAP plots exhibiting the expression patterns of *LGR5* and *TNFRSF19* (Troy) in the 10X (left) and STRT (right) datasets. Red circles highlight the positive cells. The color key from grey to blue indicates low to high expression levels, respectively.

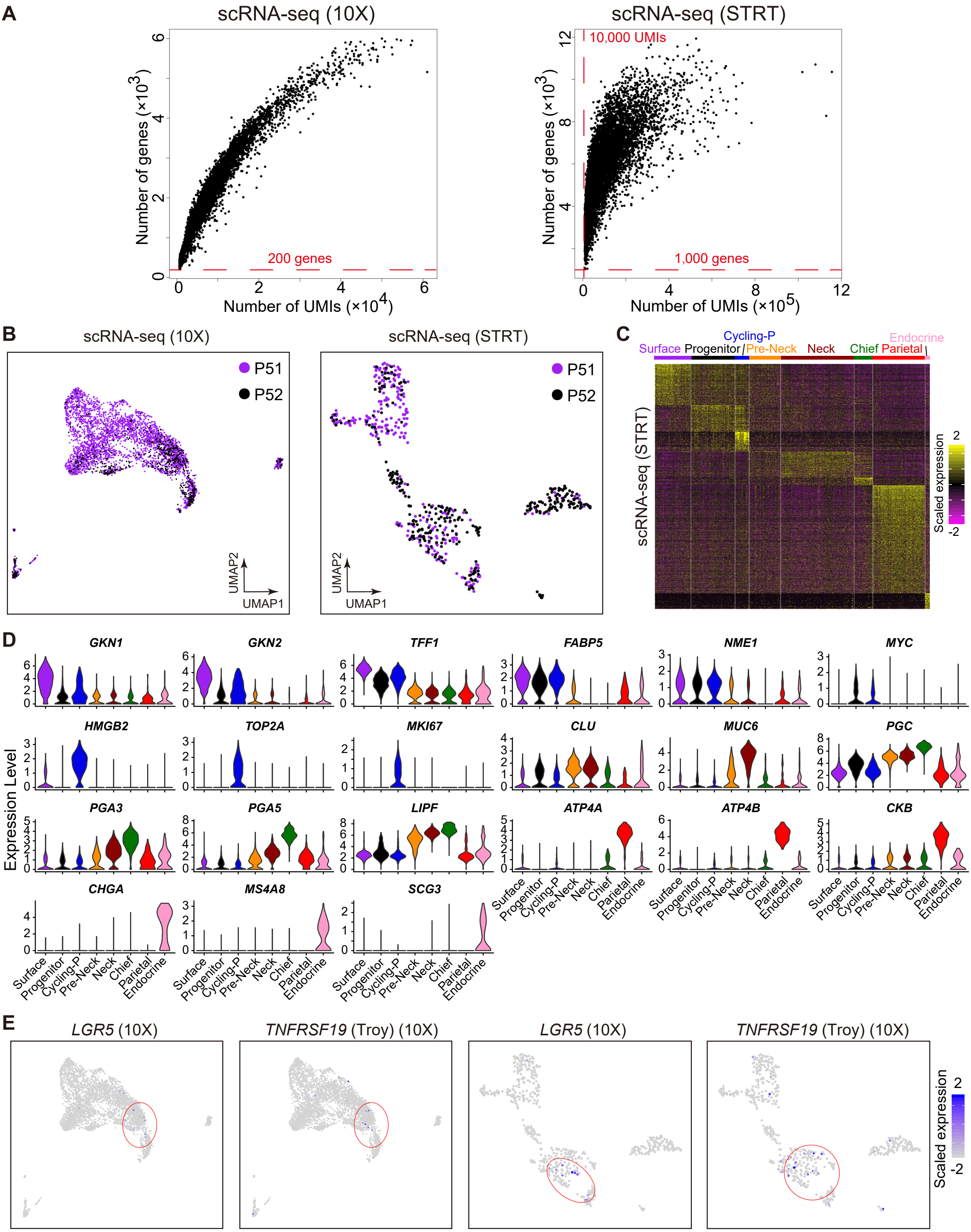
A
scRNA-seq (10X)
scRNA-seq (STRT)
Number of genes (×10^3)
Number of UMIs (×10^4)
Number of UMIs (×10^5)
200 genes
10,000 UMIs
1,000 genes
B
P51
P52
UMAP1
UMAP2
C
Surface
Progenitor
Cycling-P
Pre-Neck
Neck
Chief
Parietal
Endocrine
scRNA-seq (STRT)
Scaled expression
D
Expression Level
GKN1
GKN2
TFF1
FABP5
NME1
MYC
HMGB2
TOP2A
MKI67
CLU
MUC6
PGC
PGA3
PGA5
LIPF
ATP4A
ATP4B
CKB
CHGA
MS4A8
SCG3
E
LGR5 (10X)
TNFRSF19 (Troy) (10X)
Scaled expression

Fig. S2. Clustering of spatial transcriptomics data

(A) H&E staining of P54 sample section used for spatial transcriptomics.

(B) The expression pattern of *MUC5AC* and *MUC6* in P54 spots. The color key from blue to red indicates low to high expression levels, respectively.

(C) Heatmap showing DEGs of each cluster in P53 spatial transcriptomics. The color key from purple to yellow indicates low to high expression levels, respectively.

(D) Dotplots exhibiting representative marker genes in P53 spatial transcriptomics. The color key from white to purple indicates low to high expression levels, respectively. The circle size indicates the percentage of cells expressing a certain gene.

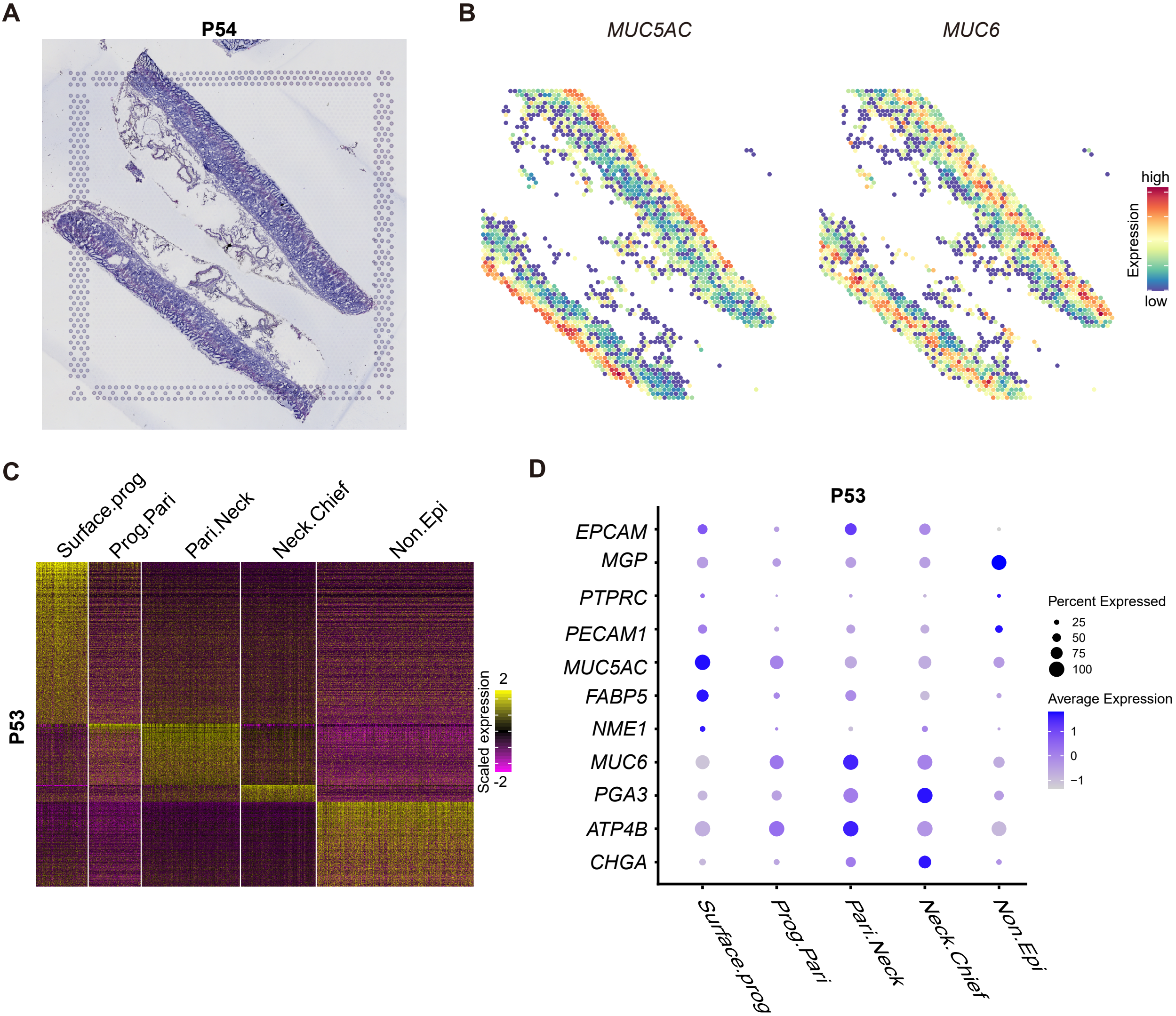

A
P54
B
MUC5AC
MUC6
high
Expression
low
C
Surface.prog
Prog.Pari
Pari.Neck
Neck.Chief
Non.Epi
P53
Scaled expression
2
-2
D
P53
EPCAM
MGP
PTPRC
PECAM1
MUC5AC
FABP5
NME1
MUC6
PGA3
ATP4B
CHGA
Percent Expressed
25
50
75
100
Average Expression
1
0
−1
Surface.prog
Prog.Pari
Pari.Neck
Neck.Chief
Non.Epi

Fig. S3. Gene expression landscapes of human ileal epithelium and differentiation trajectory of human corpus epithelium

(A) UMAP visualization of human adult ileal epithelium. Cells are colored by patient information (left) and cell type information (right). TA: transit-amplifying; Mcell: microfold cell.

(B) Heatmaps displaying DEGs within each cluster. For the convenience of visualization, clusters exceeding 100 cells were down sampled to 100 cells. The color key from purple to yellow indicates low to high expression levels, respectively.

(C) Dotplot displaying the expression levels of representative marker genes of each cluster. Spot size indicates the percentage of cells expressing the gene within each cluster and color intensity denotes the expression levels of the gene.

(D) Four differentiation trajectories of human corpus stem/progenitor cells (left). On the right are heatmaps exhibiting the top 100 genes relevant to each trajectory. The color key from blue to red indicates low to high expression levels, respectively.

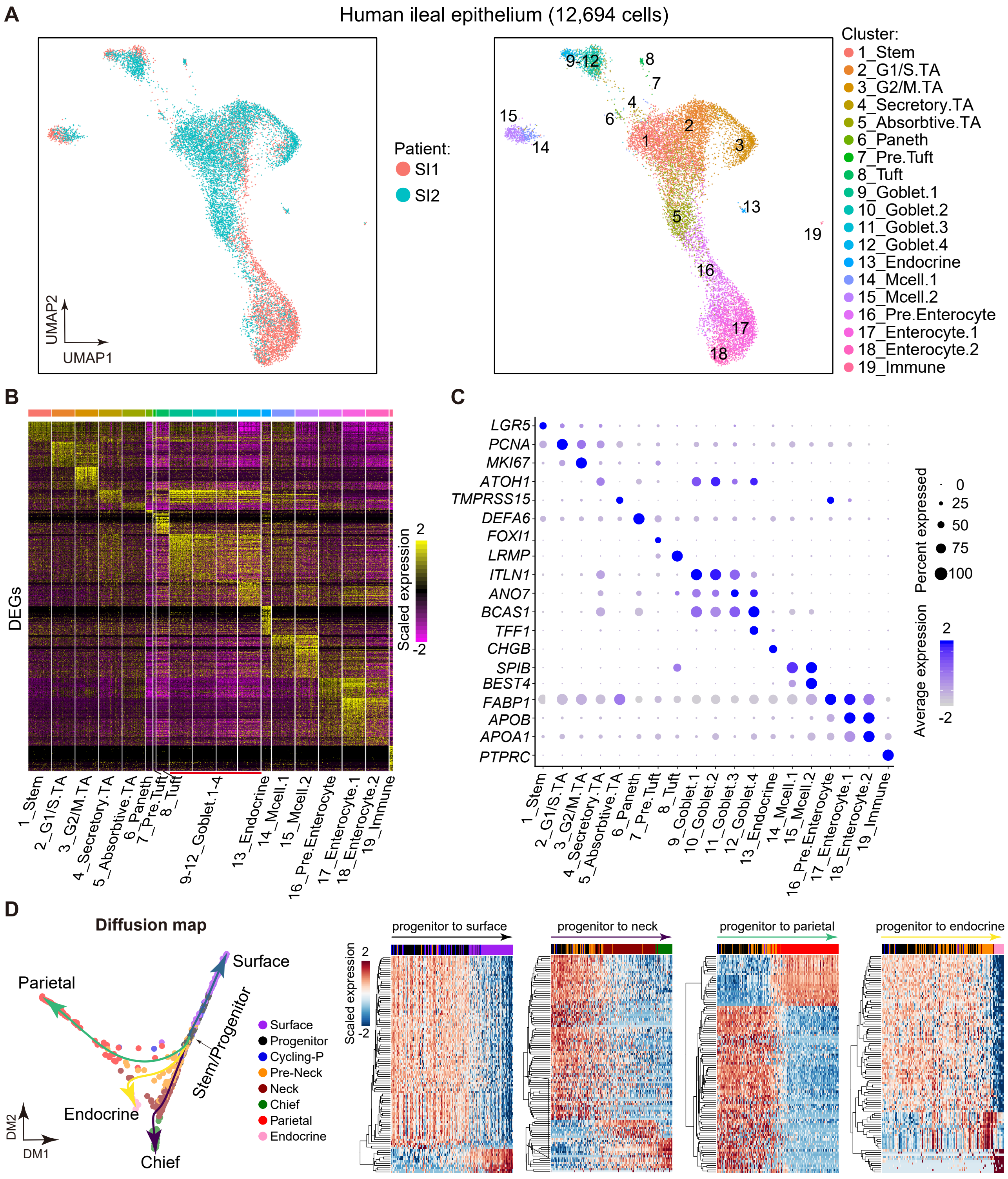
A
Human ileal epithelium (12,694 cells)
UMAP2
UMAP1
Patient:
SI1
SI2
Cluster:
1_Stem
2_G1/S.TA
3_G2/M.TA
4_Secretory.TA
5_Absorbtive.TA
6_Paneth
7_Pre.Tuft
8_Tuft
9_Goblet.1
10_Goblet.2
11_Goblet.3
12_Goblet.4
13_Endocrine
14_Mcell.1
15_Mcell.2
16_Pre.Enterocyte
17_Enterocyte.1
18_Enterocyte.2
19_Immune
B
DEGs
Scaled expression
9-12_Goblet.1-4
C
LGR5
PCNA
MKI67
ATOH1
TMPRSS15
DEFA6
FOXI1
LRMP
ITLN1
ANO7
BCAS1
TFF1
CHGB
SPIB
BEST4
FABP1
APOB
APOA1
PTPRC
Percent expressed
Average expression
D
Diffusion map
Surface
Parietal
Stem/Progenitor
Endocrine
Chief
DM2
DM1
Progenitor
Cycling-P
Pre-Neck
Neck
progenitor to surface
progenitor to neck
progenitor to parietal
progenitor to endocrine

Fig. S4. Gene ontology analysis of DEGs identified in gene knockdown experiments

(A) Heatmap exhibiting the DEGs of *MYC*, *FABP5* and *NME1* knockdown group compared with negative control siRNA group (48h after transfection) (left). The right panel is the heatmap exhibiting the upregulated DEGs in the STRT scRNA-seq dataset. The color key from purple to yellow indicates low to high expression levels, respectively.

(B) Top enriched terms using the upregulated (left) and downregulated (right) DEGs of MYC siRNA group compared with negative control siRNA group.

(C) Top enriched terms using the upregulated (left) and downregulated (right) DEGs of FABP5 siRNA group compared with negative control siRNA group.

(D) Top enriched terms using the upregulated (left) and downregulated (right) DEGs of NME1 siRNA group compared with negative control siRNA group.

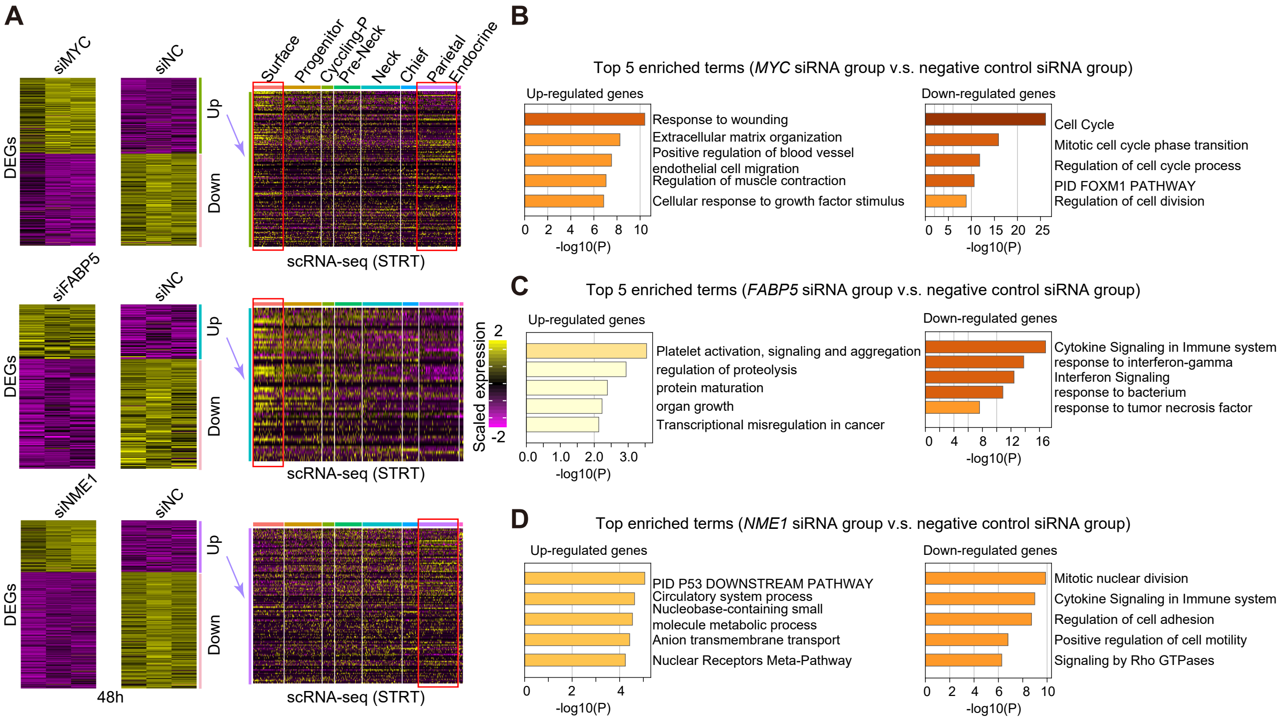

A
siMYC
siNC
DEGs
Up
Down
Surface
Progenitor
Cyccling-P
Pre-Neck
Neck
Chief
Parietal
Endocrine
scRNA-seq (STRT)
siFABP5
siNC
DEGs
Up
Down
scRNA-seq (STRT)
Scaled expression
2
-2
siNME1
siNC
DEGs
Up
Down
48h
scRNA-seq (STRT)
B
Top 5 enriched terms (MYC siRNA group v.s. negative control siRNA group)
Up-regulated genes
Response to wounding
Extracellular matrix organization
Positive regulation of blood vessel endothelial cell migration
Regulation of muscle contraction
Cellular response to growth factor stimulus
0 2 4 6 8 10
-log10(P)
Down-regulated genes
Cell Cycle
Mitotic cell cycle phase transition
Regulation of cell cycle process
PID FOXM1 PATHWAY
Regulation of cell division
0 5 10 15 20 25
-log10(P)
C
Top 5 enriched terms (FABP5 siRNA group v.s. negative control siRNA group)
Up-regulated genes
Platelet activation, signaling and aggregation
regulation of proteolysis
protein maturation
organ growth
Transcriptional misregulation in cancer
0.0 1.0 2.0 3.0
-log10(P)
Down-regulated genes
Cytokine Signaling in Immune system
response to interferon-gamma
Interferon Signaling
response to bacterium
response to tumor necrosis factor
0 4 8 12 16
-log10(P)
D
Top enriched terms (NME1 siRNA group v.s. negative control siRNA group)
Up-regulated genes
PID P53 DOWNSTREAM PATHWAY
Circulatory system process
Nucleobase-containing small molecule metabolic process
Anion transmembrane transport
Nuclear Receptors Meta-Pathway
0 2 4
-log10(P)
Down-regulated genes
Mitotic nuclear division
Cytokine Signaling in Immune system
Regulation of cell adhesion
Positive regulation of cell motility
Signaling by Rho GTPases
0 2 4 6 8 10
-log10(P)

Fig. S5. The function of *FABP5* and *NME1* for gastric cancers

(A) RNAscope staining for *FABP5*, *NME1* and *MKI67* on gastric tumor sample from another patient. Scale bar: 1 mm.

(B) FACS analysis of representative control sample for apoptosis experiment.

(C) FACS analysis of representative gastric cancer cell line for apoptosis experiment.

(D) FACS analysis of representative gastric cancer cell line for cell cycle experiment.

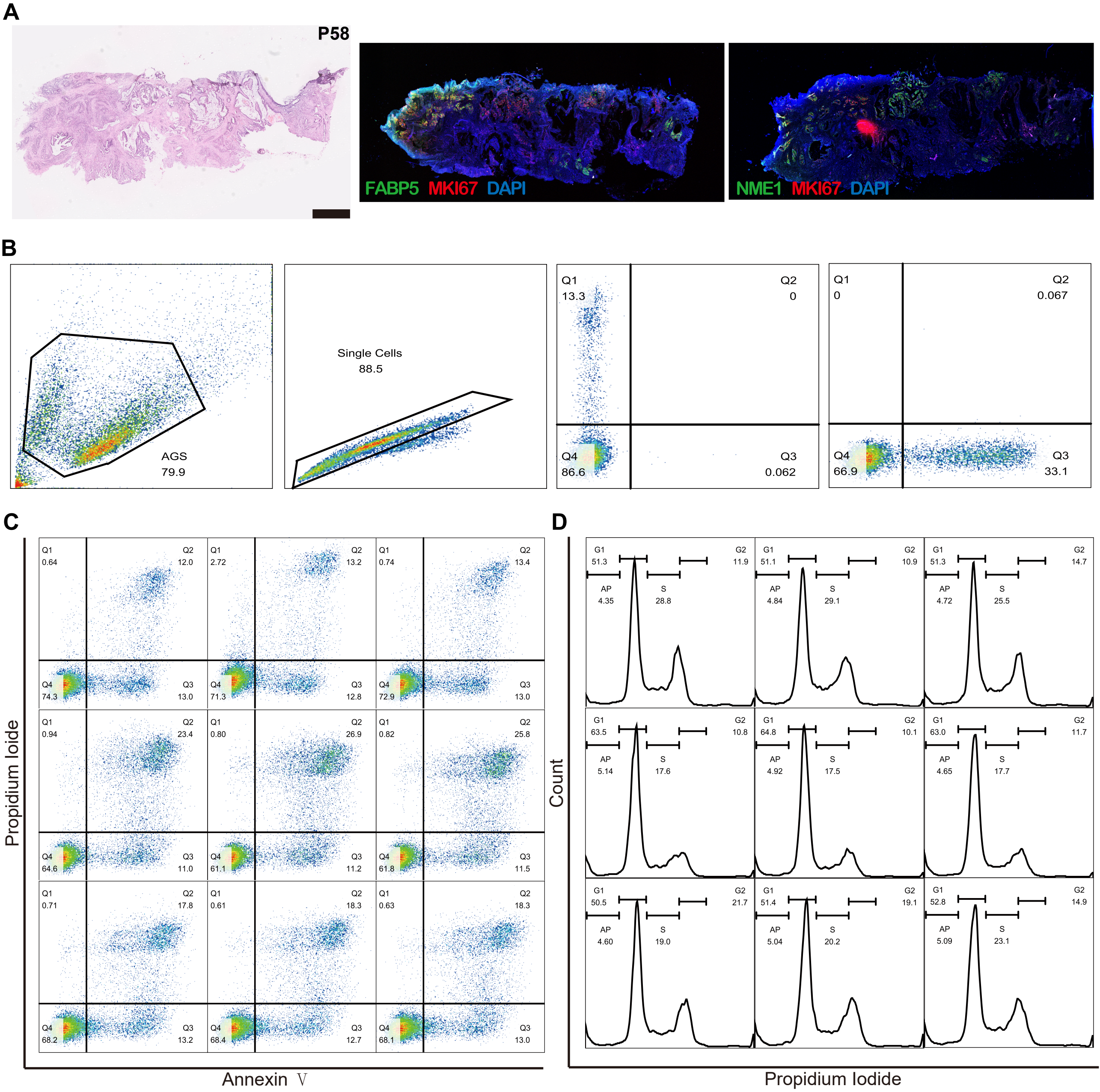
A
P58
FABP5 MKI67 DAPI
NME1 MKI67 DAPI
B
AGS
79.9
Single Cells
88.5
Q1
13.3
Q2
0
Q4
86.6
Q3
0.062
Q1
0
Q2
0.067
Q4
66.9
Q3
33.1
C
Q1 0.64 Q2 12.0 Q4 74.3 Q3 13.0
Q1 2.72 Q2 13.2 Q4 71.3 Q3 12.8
Q1 0.74 Q2 13.4 Q4 72.9 Q3 13.0
Q1 0.94 Q2 23.4 Q4 64.6 Q3 11.0
Q1 0.80 Q2 26.9 Q4 61.1 Q3 11.2
Q1 0.82 Q2 25.8 Q4 61.8 Q3 11.5
Q1 0.71 Q2 17.8 Q4 68.2 Q3 13.2
Q1 0.61 Q2 18.3 Q4 68.4 Q3 12.7
Q1 0.63 Q2 18.3 Q4 68.1 Q3 13.0
Propidium Iodide
Annexin Ⅴ
D
G1 51.3 G2 11.9 AP 4.35 S 28.8
G1 51.1 G2 10.9 AP 4.84 S 29.1
G1 51.3 G2 14.7 AP 4.72 S 25.5
G1 63.5 G2 10.8 AP 5.14 S 17.6
G1 64.8 G2 10.1 AP 4.92 S 17.5
G1 63.0 G2 11.7 AP 4.65 S 17.7
G1 50.5 G2 21.7 AP 4.60 S 19.0
G1 51.4 G2 19.1 AP 5.04 S 20.2
G1 52.8 G2 14.9 AP 5.09 S 23.1
Count
Propidium Iodide

Fig. S6. Genomic tracks exhibiting the accessibility of representative marker peaks in aggregated scATAC-seq clusters

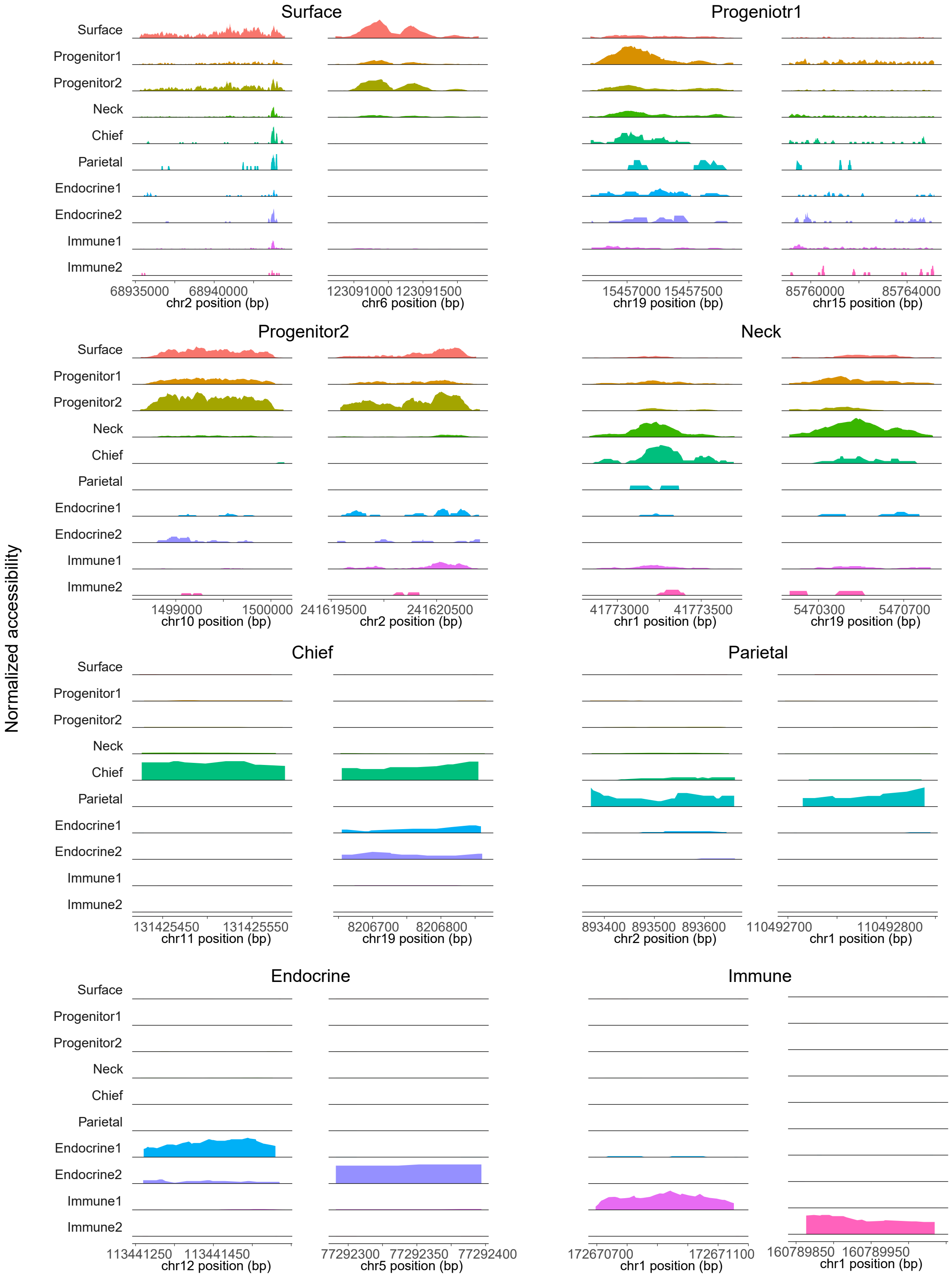
Normalized accessibility
Surface
Progeniotr1
Progenitor2
Neck
Chief
Parietal
Endocrine
Immune
Surface
Progenitor1
Progenitor2
Neck
Chief
Parietal
Endocrine1
Endocrine2
Immune1
Immune2
68935000
68940000
chr2 position (bp)
123091000 123091500
chr6 position (bp)
15457000 15457500
chr19 position (bp)
85760000
85764000
chr15 position (bp)
1499000
1500000
chr10 position (bp)
241619500
241620500
chr2 position (bp)
41773000
41773500
chr1 position (bp)
5470300
5470700
chr19 position (bp)
131425450
131425550
chr11 position (bp)
8206700
8206800
chr19 position (bp)
893400 893500 893600
chr2 position (bp)
110492700
110492800
chr1 position (bp)
113441250
113441450
chr12 position (bp)
77292300 77292350 77292400
chr5 position (bp)
172670700
172671100
chr1 position (bp)
160789850 160789950
chr1 position (bp)

Fig. S7. TF regulatory network of human gastric corpus epithelium

(A) Heatmaps exhibiting the differentially activated TFs of aggregated scATAC-seq clusters. The color key from blue to red/yellow indicates low to high deviation score, respectively.

(B) Regulatory network of cell type specific TFs targeting the top 100 DEGs of human corpus epithelium. Genes are colored by cell type information.

(C) Heatmap exhibiting the MYC regulated genes overlapped with potential target genes and corpus DEG (left), which was identified by MYC knockdown siRNA compared with negative control siRNA after 48h. On the right is the heatmap exhibiting these genes in the STRT scRNA-seq dataset. The color key from purple to yellow indicates low to high expression levels, respectively.

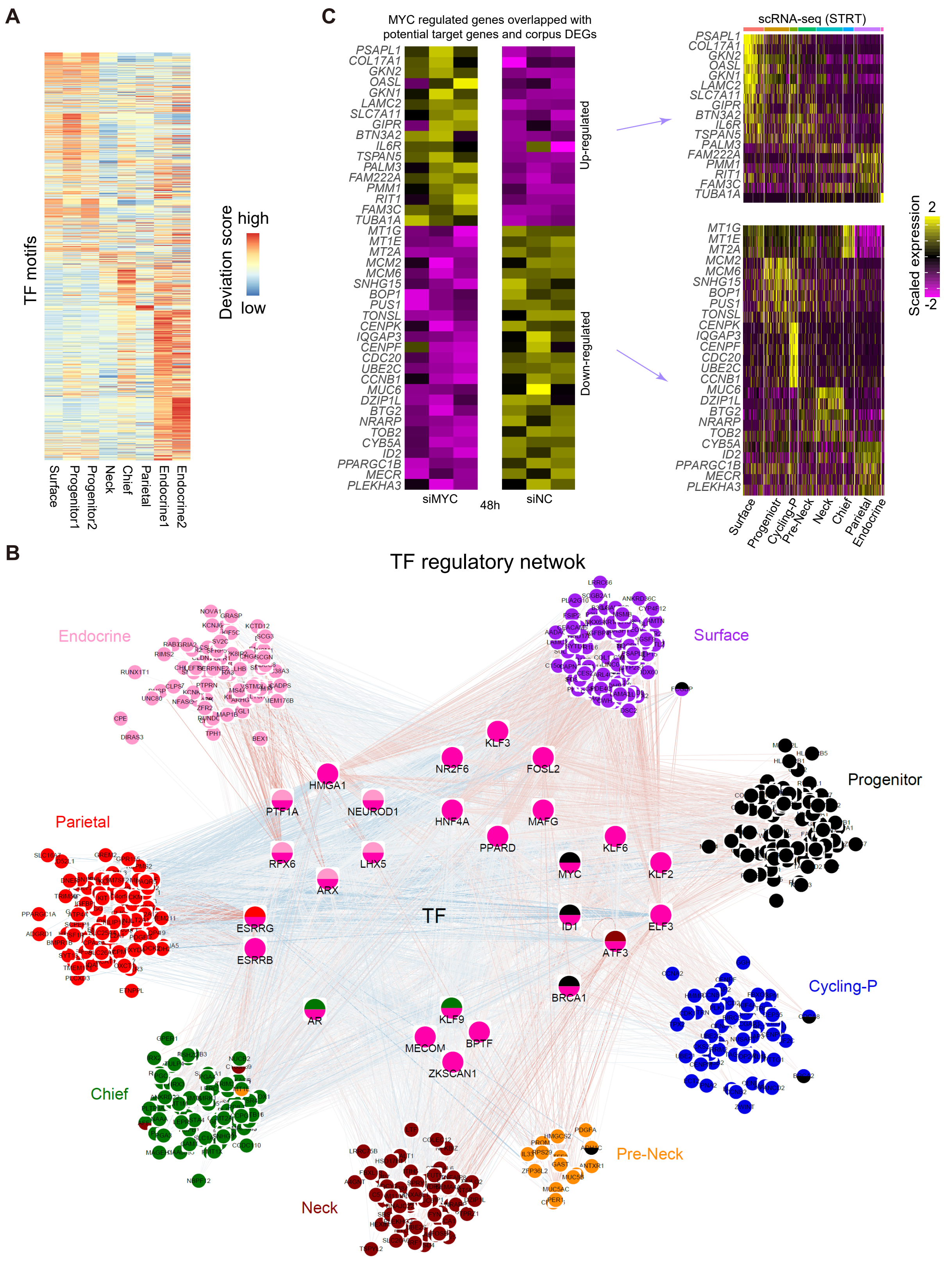
A
TF motifs
Deviation score
high
low
Surface
Progenitor1
Progenitor2
Neck
Chief
Parietal
Endocrine1
Endocrine2
C
MYC regulated genes overlapped with potential target genes and corpus DEGs
PSAPL1
COL17A1
GKN2
OASL
GKN1
LAMC2
SLC7A11
GIPR
BTN3A2
IL6R
TSPAN5
PALM3
FAM222A
PMM1
RIT1
FAM3C
TUBA1A
MT1G
MT1E
MT2A
MCM2
MCM6
SNHG15
BOP1
PUS1
TONSL
CENPK
IQGAP3
CENPF
CDC20
UBE2C
CCNB1
MUC6
DZIP1L
BTG2
NRARP
TOB2
CYB5A
ID2
PPARGC1B
MECR
PLEKHA3
siMYC
48h
siNC
Up-regulated
Down-regulated
scRNA-seq (STRT)
Scaled expression
2
-2
Surface
Progeniotr
Cycling-P
Pre-Neck
Neck
Chief
Parietal
Endocrine
B
TF regulatory netwok
Endocrine
Surface
Progenitor
Parietal
Cycling-P
Chief
Pre-Neck
Neck
TF
KLF3
NR2F6
FOSL2
HMGA1
PTF1A
NEUROD1
HNF4A
MAFG
PPARD
KLF6
RFX6
LHX5
MYC
KLF2
ARX
ESRRG
ID1
ELF3
ATF3
ESRRB
BRCA1
AR
KLF9
MECOM
BPTF
ZKSCAN1

Supplemental Table Legends

Supplemental Table 1. scRNA-seq of human gastric corpus epithelium

Supplemental Table 2. DEGs of human gastric corpus epithelium

Supplemental Table 3. Barcode information

Supplemental Table 4. Peak-to-genes result
